# Transcriptional dynamics during Heliothis zea nudivirus 1 infection in an ovarian cell line from Helicoverpa zea

**DOI:** 10.1099/jgv.0.002066

**Published:** 2025-01-13

**Authors:** Jirka Manuel Petersen, Astrid Bryon, Annie Bézier, Jean-Michel Drezen, Monique M. van Oers

**Affiliations:** 1 Laboratory of Virology, Wageningen University and Research, 6708 PB Wageningen, Netherlands; 2 Institut de Recherche sur la Biologie de l’Insecte, UMR 7261 CNRS - Université de Tours, 37200 Tours, France

**Keywords:** differential expression, *Helicoverpa zea*, immune response, *Nudiviridae*, ovarian cells, RNA-seq

## Abstract

Nudiviruses (family *Nudiviridae*) are double-stranded DNA viruses that infect various insects and crustaceans. Among them, Heliothis zea nudivirus 1 (HzNV-1) represents the rare case of a lepidopteran nudivirus inducing a sexual pathology. Studies about molecular pathological dynamics of HzNV-1 or other nudiviruses are scarce. Hence, this study aims to provide a transcriptomic profile of HzNV-1 in an ovary-derived cell line of *Helicoverpa zea* (HZ-AM1), during early (3, 6 and 9 h post-infection) and advanced (12 and 24 h post-infection) stages of infection. Total RNA was extracted from both virus- and mock-infected cells, and RNA-seq analysis was performed to examine both virus and host transcriptional dynamics. Hierarchical clustering was used to categorize viral genes, while differential gene expression analysis was utilized to pinpoint host genes that are significantly affected by the infection. Hierarchical clustering classified the 154 HzNV-1 genes into four temporal phases, with early phases mainly involving transcription and replication genes and later phases including genes for virion assembly. In addition, a novel viral promoter motif was identified in the upstream region of early-expressed genes. Host gene analysis revealed significant upregulation of heat shock protein genes and downregulation of histone genes. The identification of temporal patterns in viral gene expression enhances the molecular understanding of nudivirus pathology, while the identified differentially expressed host genes highlight the key pathways most hijacked by HzNV-1 infection.

## Data availability

The raw reads of the RNA-seq experiment are reposited in the Sequence Read Archives (SRA) under the Bioproject number PRJNA1177232 (accessions from SRX26483606 to SRX26483629).

## Importance

Among the members of the order *Lefavirales*, nudiviruses have an exceptionally broad host range and diverse pathological dynamics, with new members being discovered frequently in insects and crustaceans. As nudiviruses are economically significant pathogens of invertebrates, these viruses warrant more in-depth studies to understand their molecular pathology and gene functions, but such studies are currently underrepresented. Our study offers insights into the gene expression profile of a member of the genus *Betanudivirus* with described potential as a biocontrol agent and provides implications on how nudiviruses might modulate the cellular machinery and integrity of their host. These findings may pave the way for identifying targets to combat damaging nudivirus infections in crustaceans and insect rearing or improve nudivirus efficacy in biocontrol applications.

## Introduction

Nudiviruses (family *Nudiviridae*) are insect- and crustacean-infecting viruses that share their taxonomic order (*Lefavirales*) with baculoviruses (*Baculoviridae*), hytrosaviruses (*Hytrosaviridae*) and the recently proposed family Filamentoviridae [1]. The large circular, dsDNA genomes that nudiviruses have in common with the other members of the *Lefavirales* range from ~96 to ~231 kbp among known nudivirus species. The rod-shaped virions of nudiviruses vary in length from ~120 to ~414 nm, while diameters range from ~30 to ~80 nm (reviewed by [2]). In most cases, these nudiviral virions are perorally transmitted through ingestion of contaminated excrements or carcasses, e.g. through cannibalism [3,4], but also sexual and transovarial transmissions have been observed for a member of the genus *Betanudivirus* [5,6]. The genus *Betanudivirus* is one of the four officially recognized genera of *Nudiviridae* and is represented by two isolates belonging to the same virus species, *Betanudivirus hezeae* [7]. These two virus isolates, Heliothis zea nudivirus 1 (HzNV-1) and Helicoverpa zea nudivirus 2 (HzNV-2), are currently the only known lepidopteran-infecting nudiviruses, and they can be mutually distinguished by particular characteristics. For instance, HzNV-2 can infect larvae of its natural host *Helicoverpa zea* (*Lepidoptera*: *Noctuidae*) and cause malformations in the reproductive organs of adult moths [8], whereas HzNV-1 was originally found in an *H. zea *(formerly *Heliothis zea) *derived cell culture and has lost the ability to naturally infect insects [9,10]. Nevertheless, HzNV-1 can infect a wide range of lepidopteran cell lines, including IPLB-1075 and HZ-AM1 (*H. zea*), IPLB-Sf-21 (*Spodoptera frugiperda*), IPLB-65Z (*Lymantria dispar*) and TN-368 (*Trichoplusia ni*), causing lytic infections with cytopathogenic effects (CPE) [10,12].

The most well-studied nudivirus, Oryctes rhinoceros nudivirus (OrNV), a member of the *Alphanudivirus* genus, was the subject of several transcription profiling studies [13,15], which provided the first insights into nudivirus gene expression. However, these studies focused on single infection time points in *Oryctes rhinoceros* (*Coleoptera*: *Scarabaeidae*) populations, providing little information on the transcriptional changes over the course of a progressing nudivirus infection. On the other hand, progressive transcriptional changes have been investigated for bracoviruses, a clade of endogenous viruses found in braconid wasps, which originates from an ancestral nudivirus [16,18]. The available transcriptomic data on endogenous viruses of nudiviral origin involved in wasp parasitism provide valuable insights into the sequential expression of nudivirus core gene homologues integrated into the wasp genome during ovary development [19,21]. However, due to their unconventional life cycle, such bracoviral gene expression profiles in the wasp cannot simply be extrapolated to the situation in cells infected by exogenous nudiviruses. Nevertheless, it will be valuable to compare and discuss the expression pattern of pathogenic and endogenous nudivirus genes to determine to what extent these are similar.

In this study, we provide a transcriptional profile throughout early [3, 6 and 9 h post-infection (hpi)] and advanced (12 and 24 hpi) HzNV-1 infection in HZ-AM1 cells to expand our knowledge on nudivirus cytopathology. This includes expression profiling of the 154 officially annotated HzNV-1 genes to determine their temporal expression patterns. Furthermore, we identify differentially expressed genes (DEGs) in the host at the mentioned time points to get insight into the interactions of HzNV-1 with the ovarian-derived host cells. Finally, the relevance of those transcripts for the success of the nudivirus infection versus the host immune response will be discussed.

## Methods

### Insect cell line and virus

The main infectious agent of this study was the *Betanudivirus* HzNV-1 [9]. The nudivirus was passaged in the cell line, HZ-AM1 (originally referred to as BCIRL-HZ-AM1), which is derived from the ovarian tissue of *H. zea* [22]. The HZ-AM1 cells were grown at 28 °C in HyClone™ CCM3 medium (Cytiva) supplemented with 5% FBS (Life Technologies) and 50 µg ml^−1^ gentamicin (Gibco). Fresh HzNV-1 inoculum was produced from a frozen stock stored at −80 °C (Passage zero (P0) isolate) that was provided by Professor Yueh-Lung Wu (Department of Entomology, National Taiwan University, Taipei, Taiwan) in 2020, by collecting and filtrating (0.45 µm pore size) the supernatant P0-infected HZ-AM1 cells 2 days post-infection. This procedure was repeated over three passages of HZ-AM1 subcultures and the acquired HzNV-1 inoculum was hence designated as the P3 isolate. The virus titre of the P3 isolate was determined via endpoint dilution assay [23].

### Infection of HZ-AM1 cells with HzNV-1 for transcriptome analysis

HZ-AM1 cells were first grown in 75 cm^2^ flasks (T75) as described above. Fully grown 12 ml cultures of HZ-AM1 cells were detached and resuspended in the medium to create a homogenous cell suspension. The cell suspension was then evenly distributed over up to twelve 25 cm^2^ flasks (T25) in 1 ml aliquots and supplemented each with 3 ml of CCM3 medium as described above. The T25 flasks with HZ-AM1 cells were then left to grow for 24 h at 28 °C. After removal of the medium from the attached cells, triplicates were incubated for 45 min at 28 °C with the P3 isolate of HzNV-1 at a multiplicity of infection (MOI) of 5, while control groups were incubated in triplicates with virus-free medium. After virus or mock incubation, the supernatant (corresponding to virus inoculum or virus-free medium, respectively) was removed, and 4 ml of fresh CCM3 medium was added. This procedure was carried out for two separate infection experiments. In the first experiment, virus-infected cell cultures were harvested for RNA extraction at 3, 6 and 9 hpi, each in triplicate. Mock-infected cells, serving as the control, were harvested at 0 hpi, also in triplicate. The second experiment involved both virus- and mock-infected cells, which were harvested at 12 and 24 hpi for RNA extraction, with all samples handled in triplicates.

### Whole RNA extraction

The virus- and mock-infected cells were harvested at the defined time points for RNA extraction by removing the medium and washing them once in 1 × PBS. After PBS removal, 1 ml of TRIzol^®^ (Thermo Fisher Scientific) was quickly added to the cells in the T25 flasks. The cells were detached with a bent glass pipette and at the same time resuspended in the TRIzol^®^. A micropipette was then used to homogenize the cell–TRIzol suspension by pipetting up and down. Next, the 1 ml cell–TRIzol suspension was transferred to a 1.5-ml reaction tube and incubated for 5 min at room temperature. After the addition of 200 μl chloroform to each sample, the suspension was mixed by vigorously shaking the tube. The sample was again incubated at room temperature for 5 min and subsequently centrifuged for 15 min at 12 000 ***g*** at 4 °C. The upper colourless aqueous phase containing the RNA was then transferred to a new 1.5-ml tube and mixed with 500 µl isopropanol. The sample was incubated for 10 min at room temperature and subsequently centrifuged at 12 000 ***g*** for 10 min at 4 °C. The supernatant was removed, and the RNA pellet was washed in 1 ml 75% ethanol by a brief stir on the vortex mixer. Afterwards, the sample was centrifuged for 5 min at 7500 ***g*** at 4 °C, and the ethanol was carefully removed from the pellet. To remove residual ethanol, the lid of the tube was left open for 5 to 10 min at room temperature. Finally, the air-dried pellet was resuspended in 30–50 μl RNAse-free water by flicking, and the RNA concentration was determined spectrophotometrically.

### RNA sequencing and DEG analysis

**Fig. 1.**
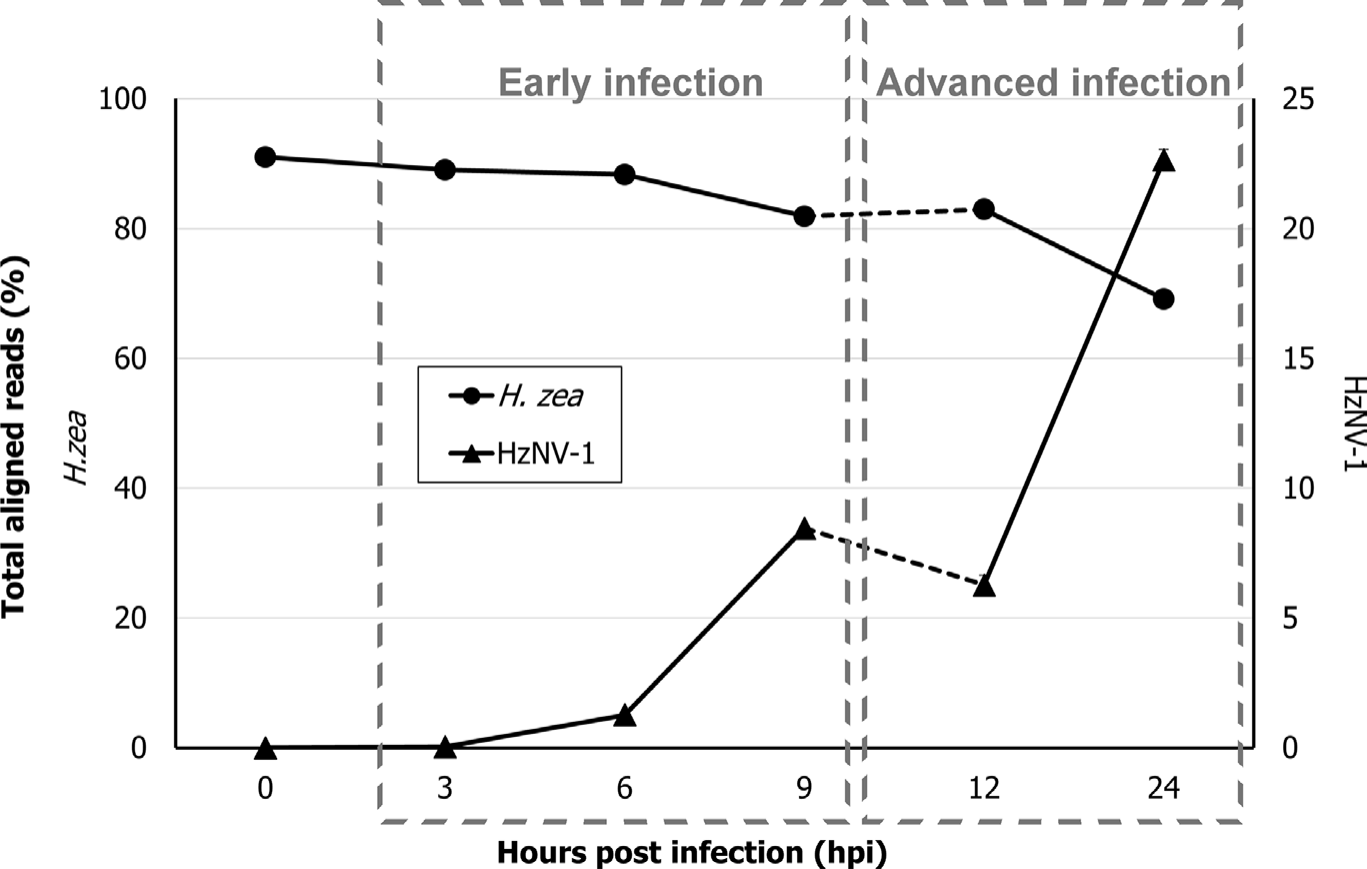
Time-dependent changes of mRNA reads from *H. zea* and HzNV-1 in percentages. The percentages of total reads mapped to the *H. zea* genome (triplicate average percentage of mRNA reads) are displayed as circles (left *y*-axis), and the average percentages of the total HzNV-1 reads are displayed as triangles (right *y*-axis). The dashed lines between 9 and 12 hpi emphasize that those time points are from two distinct experiments (early infection, 0, 3, 6 and 9 hpi; advanced infection, 12 and 24 hpi).

All RNA samples were processed for sequencing according to the TruSeq stranded total RNA Ribo-Zero H/M/R Gold protocol (Illumina) and yielded RNA concentrations ranging from 3.33 to 20.90 ng µl^−1^ after library preparation. The resulting library was sequenced by Macrogen Inc. (Seoul, South Korea) using the NovaSeq6000 sequencing system to generate paired-end reads of 151 bp. The raw, paired-end reads were filtered and trimmed with fastp v0.23.2 [24] under default settings, and the clean reads were used for further analyses. Host and virus reference genomes were indexed by using the ‘hisat2-build’ command in HISAT2 v2.2.1 [25]. In the case of *H. zea*, two separate files containing lists of previously extracted exons and splice sites were provided to the hisat2-build command using the --exon and --ss parameters, along with the reference genome file. Afterwards, the filtered paired-end reads were aligned to the indexed *H. zea* reference genome (ilHelZeax1.1, GenBank: GCA_022581195.1) using HISAT2. The alignment was performed with the -x option to specify the genome index and the −1 and −2 options to provide the paired-end read files. The output was saved as a SAM file using the -S parameter, while unaligned read pairs were captured with the --un-conc option to save them into separate files. The unmapped reads were subsequently mapped with HISAT2 to the indexed HzNV-1 genome using the -x option to specify the genome index and the −1 and −2 options to assign the unmapped paired-end reads, resulting in the generation of another SAM file [26]. The obtained SAM files were sorted and converted into BAM files with SAMtools v1.15.1 [27]. Percentages of reads that aligned with the respective reference genome were extracted from the BAM files by using the ‘flagstat’ command from SAMtools (Fig. 1). The obtained BAM files were used as input for the transcript assembly tool StringTie [28] while specifying the -e option to estimate transcript abundances and the -B option to create a Ballgown [29] table that stores the transcript-level abundance estimates, as well as including the --rf option for TruSeq stranded data. The resulting files were then submitted to the python script ‘prepDE.py’ (https://github.com/gpertea/stringtie/blob/master/prepDE.py, accessed on 10 June 2022) to convert the host and virus read counts to their respective CSV files with read count matrices for genes and transcripts. Due to the small sample sizes in our experiments, we used the R package limma v3.50.3 [30] to test for differential host gene expression during early (3, 6 and 9 hpi) and advanced (12 and 24 hpi) HzNV-1 infection in HZ-AM1 cells. The early infection time points (3, 6 and 9 hpi) were all compared to a mock-infected sample at 0 hpi, while the two advanced infection time points (12 and 24 hpi) were compared to the respective mock-infected time points at 12 and 24 hpi. Host genes with absolute read counts below six were considered lowly expressed and filtered out, while the remaining reads were normalized using the limma-voom method, which includes quantile normalization to adjust for distributional differences [31]. For HzNV-1, the read counts for each sample were then normalized based on the total number of trimmed and filtered reads per sample (including both host and viral reads) and used for downstream analyses. Host genes were considered differently expressed if their false discovery rate (FDR) values were lower than 0.05, and changes in their gene expression levels exceeded a log2 fold change (log2FC) = log2(1.5), accounting for an increase or reduction in gene expression by 50%. The proteome of *H. zea* was annotated with the ‘Annotate Your Proteome’ function of the STRING: functional protein association networks webtool v12.0 [32]. The inferred proteome was created under the string taxid ‘STRG0A69IJE’ and yielded a list of 23 696 proteins, which is publicly accessible. This proteome was then supplied to the ‘Multiple proteins’ function of STRING, together with the list of the 570 different host genes that were differently expressed during HzNV-1 infection. After inferring the protein network, protein clusters were determined by choosing the Markov Cluster Algorithm (MCL) clustering option in the ‘Clusters’ tab and specifying an MCL inflation parameter of 1.5. The ‘Exports’ tab was used to extract the functional enrichment terms with their assigned proteins. The protein networks were then imported from the STRING webtool into Cytoscape v3.10.2 [33], where network layouts were tailored and nodes (proteins) coloured based on the log2FC of their corresponding genes. Venn diagrams showing the conservation among DEGs of different infection time points were created with the vennDiagram() function of the aforementioned limma R package. Additionally, host DEGs were visualized in time point-specific volcano plots in Fig. S1 (available in the online Supplementary Material 1).

### HzNV-1 gene transcription profiling

**Fig. 2.**
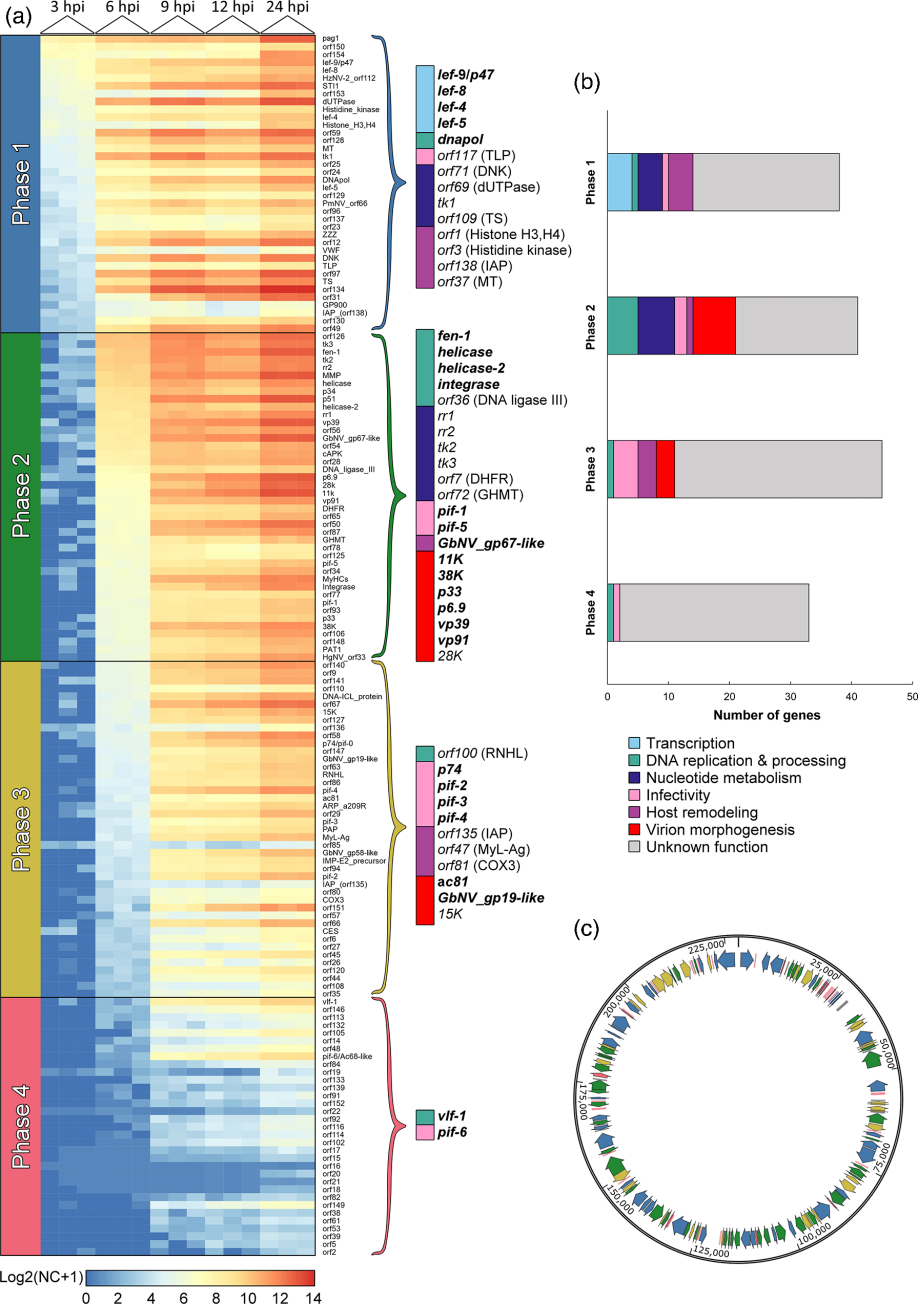
Gene expression profile of HzNV-1 genes over time. (**a**) Heatmap showing the log2 transformed, normalized counts (NC) from each triplicate of the respective time point shown in the indicated rows. Gene expression levels range from blue (low expression) to red (high expression). The 154 viral ORFs (excluding *pag1* and PAT1) were grouped into 37 Phase 1 genes, 41 Phase 2 genes, 43 Phase 3 genes and 33 Phase 4 genes. Tall curly brackets emphasize notable HzNV-1 genes from each phase (nudiviral core genes written in bold), with their predicted protein annotations in parentheses. (**b**) Stacked bar chart illustrates the proportional distribution of emphasized HzNV-1 genes across the four temporal classes (Phases 1–4) according to their respective functions. (**c**) HzNV-1 genes distributed along the genome, colour-coded according to the phase to which they are appended, based on our clustering approach.

Hierarchical clustering, using the R package pheatmap (v1.0.12) [34] function with complete linkage and Euclidean distance, was applied to cluster the normalized viral read counts, enabling the assignment of transcriptional patterns to the HzNV-1 genes. Instead of performing hierarchical clustering on the read counts of all time points, we selected two time points (3 and 6 hpi) to elucidate patterns in transcription initiation. For this approach, the viral read counts of the 3 hpi samples were first visualized with pheatmap, and the optimal number of clusters was determined using the NbClust v3.0.1 R package [35]. This led to the identification of 2 as the optimal number of clusters to distinguish between expressed (Phase 1) and non-expressed genes at this earliest time point of infection. The analysis was continued using the same R tools for the 6 hpi time point, first excluding the Phase 1 genes. Under these conditions, most of the NbClust indices returned 3 as the optimal number of clusters for the corresponding read counts, allowing the remaining three gene clusters (Phases 2–4) to be inferred. A figure showing the two different heatmaps of 3 and 6 hpi and their respective clusters can be found in (Supplementary Material 1) (Fig. S2). Finally, a heatmap with the data from all samples (3 to 24 hpi) was inferred, and genes were sorted based on the previously determined clusters (Fig. 2a). For comparison, a heatmap inferred from the normalized HzNV-1 gene counts covering all five time points is provided in (Supplementary Material 1) (Fig. S3). The functional distribution of significant HzNV-1 genes from Phase 1 to Phase 4 was visualized as a stacked bar chart (Fig. 2b). The locations of the Phase 1 to Phase 4 genes in the HzNV-1 genome were visualized using SnapGene software v7.1 (Fig. 2c).

### Promoter analysis

Baculovirus-associated promoter motifs have been previously identified in HzNV-1, including the late promoter [ATG] TAAG and the early TATA box promoters TATA [A/T] T [A/T] [26]. In this study, we aimed to search for novel HzNV-1 promoter sequences and test their congruence with our RNA-seq data. We therefore manually copied the 300-bp-upstream nucleotide sequences of each HzNV-1 gene from SnapGene into a text file, which was then used as input to perform a promoter analysis using the MEME Suite 5.5.4 tools [36], with the following settings: -dna (DNA alphabet), -zoops (zero or one occurrence per sequence), -minw 6, -maxw 20 (size of the motifs between 6 and 20 bp) and -nmotifs 4 (search for four different motifs). The significant enrichment of motifs detected by MEME in HzNV-1 was further validated using the Analysis of Motif Enrichment (AME) tool [37]. The 300-bp-upstream nucleotide sequences from genes of nudiviruses belonging to different genera than HzNV-1 (OrNV: *Alphanudivirus*; Callinectes sapidus nudivirus, CsNV: *Gammanudivirus*; Tipula oleracea nudivirus, ToNV: *Deltanudivirus*; Franciscoloa pallida nudivirus, FrpNV: unclassified; Cuculoecus africanus nudivirus, CafNV: unclassified) and the baculovirus Autographa californica multiple nucleopolyhedrovirus (AcMNPV) were used as control sequences. The control sequences of each virus were supplied to individual AME analyses together with the 300-bp-upstream nucleotide sequences from HzNV-1, along with the identified motifs by MEME. Each AME analysis was run under the default parameters (Fisher’s exact test). The Tomtom tool [38] was used with default parameters to identify binding site profiles that matched the enriched motifs, which were previously derived from the non-redundant JASPAR 2022 core DNA database. Finally, the Find Individual Motif Occurrences (FIMO) tool [39] was used to search for occurrences of the enriched motifs in the 500-bp-upstream nucleotide sequences of all protein-coding *H. zea* genes.

### Prediction of transcription start sites in the HzNV-1 genome

Transcription start sites (TSS) of HzNV-1 were predicted using the R package TSSr [40] by following the standard workflow (https://github.com/Linlab-slu/TSSr, accessed on 19 April 2024) up to the annotation of TSS clusters (TCs). For this purpose, RNA-seq data from 15 paired-end BAM files representing short-reads mapped to the HzNV-1 genome at different time points post-infection (3, 6, 9, 12 and 24 hpi) were used. Specifically, the BAM files were loaded into R and used to create a TSSr object with the new() function. The TSSr object was then used as input for the gets() function to identify the genomic coordinates of transcription start sites. Biological replicates of each time point were merged with the mergeSamples() function to reduce variability. Identified TSS were filtered and normalized using a Poisson distribution under default settings and then clustered into TCs with the clusterTSS() function using the ‘peakclu’ method with a peak distance of 50 bp. Consensus clusters were determined with the consensusCluster() function under default settings, and these clusters were annotated with the annotateCluster() function. Adjustments were made to the parameters of the annotateCluster() function to account for the compact nature of the viral genome: ‘upstream’ was set between 130 and 200, ‘upstreamOverlap’ was set to 20 and ‘downstream’ was set between 0 and 100 to account for genes whose TSS localized downstream of their start codon. The visual assessment of the HzNV-1 BAM file coverage was performed in the Geneious software v2022.2.1 and visually compared to the TSS predicted by TSSr.

## Results and discussion

### Viral reads increase while host read abundance decreases with progressing HzNV-1 infection

Total RNA was extracted from HzNV-1-infected (MOI=5) and mock-infected HZ-AM1 cells at different time points (3, 6, 9, 12 and 24 hpi) and subjected to an RNA-seq analysis to investigate transcriptional dynamics of the host and virus over the course of infection. The reads were aligned to the host genome and reads that did not align to the host genome (unmapped reads) were extracted and aligned to the HzNV-1 genome. The percentages of reads aligned to the host or the virus genome are visualized in a line graph (Fig. 1). The exact number of reads for each sample and the respective percentages of reads that aligned to the host or virus genome can be found in the supplementary files (Supplementary Material 1; Table S1).

Notably, although the RNA-seq analysis was conducted in two independent experiments (early versus advanced infection; Fig. 1; see also the ‘Methods’ section), both experiments showed a similar trend. However, the introduction of some uncertainty in the transition of aligned reads between the 9 and 12 hpi interval (early and advanced experiments, respectively) needs to be considered, given the drop of aligned virus reads at 12 hpi compared to 9 hpi. Whether this decline is attributable to an antiviral response that the virus subsequently adapts to – resulting in a recovery of viral gene expression at 24 hpi – requires further experimental validation. The percentages of reads mapped to the host (*H. zea*) and the virus (HzNV-1) represent the proportion of reads that aligned to each genome, relative to the total number of filtered and trimmed reads. For instance, at the earliest measured stage of infection (3 hpi), 19 390 reads out of a total of 54 821 348 aligned to the viral genome, representing an average of 0.04% of the total reads (sem±0.0095%). In contrast, the highest percentage of HzNV-1 aligned reads was observed at 24 hpi with 22.64% (sem±0.38%) of the total reads mapping to the viral genome. During this interval, the largest increase occurred from 3 to 6 hpi with 35 times more viral transcripts. In contrast, host-specific reads gradually plummeted from a maximum of 91.04% (sem±0.35%) at 0 hpi to a final low of 69.16% (sem±0.56%) at 24 hpi. The decreasing percentages (and absolute values) (Table S1 in Supplementary Material 1) of host-specific reads over time suggest a reduction in overall host gene expression due to infection with HzNV-1. Similar observations have been made during bracovirus gene expression [41] and baculovirus infections [42] and also in viruses that are more distantly related to nudiviruses [43]. Impairing the global gene expression of the host can help the virus obstruct immunity-associated responses, while the virus hijacks the host’s cellular apparatus and resources to fit its requirements by upregulating those host genes that are crucial for virus replication. However, further gene expression studies, incorporating normalization with a stable host reference gene, are needed to validate this proposition.

### Hierarchical clustering groups HzNV-1 genes into four temporal classes

We analysed transcriptional patterns of HzNV-1 genes by hierarchical clustering with Euclidean distance on normalized viral read counts at 3 and 6 hpi to assign distinct temporal phases to the viral genes (see the ‘Methods’ section). At 3 hpi, two gene clusters were identified, distinguishing between expressed (Phase 1) and non-expressed genes. At 6 hpi, excluding Phase 1 genes, the three remaining gene clusters were assigned (Phases 2, 3 and 4). A heatmap with the read counts of all samples (3–24 hpi) was then created, and the HzNV-1 genes were grouped based on their assigned phases and sorted from largest to smallest number of reads (mean of triplicates (Fig. 2a)). The functional distribution of the phase-associated HzNV-1 genes is depicted in a stacked bar chart (Fig. 2b), and their genomic locations in the HzNV-1 genome were displayed (Fig. 2c). Based on our hierarchical clustering approach, we were able to group the 154 HzNV-1 protein-coding genes into 37 Phase 1 genes (24.02%), 41 Phase 2 genes (24.02%), 43 Phase 3 genes (27.92%) and 33 Phase 4 genes (21.42%) (Fig. 2a). In addition, the non-coding ‘persistency-associated gene’ (*pag1*) clusters in Phase 1 and is the transcript with the highest abundance of all transcripts in this phase, which may highlight an important role in early HzNV-1 infection, in addition to an earlier reported function in persistent infections [44]. The 28 nudiviral core genes are a set of genes that can be found in the genomes of all to date sequenced nudivirus isolates, of which 21 genes are homologous to baculovirus and 16 genes are homologous to hytrosavirus core genes. These core genes display an uneven dispersion across the four temporal classes, which likely reflects their different functions in the virus life cycle. Nudiviral core genes from Phase 1 include the transcription-associated late expression factors *lef-4*, *lef-5*, *lef-8* and *lef-9*/*p47* and the DNA polymerase, *dnapol* (Fig. 2a).

In other *Lefavirales* members and in bracoviruses, the *lef* genes are early expressed genes transcribed by the RNA polymerase of the infected cell essential for both late gene expression and virus replication [45,48], whereas the DNA polymerases of baculoviruses and hytrosaviruses have also previously been described as early genes [48,49], as they are required for the viral DNA replication. Unlike core genes, accessory genes are genes that are frequently but not universally present in the genomes of members within a clade. The accessory genes encoding for a dUTPase (*orf69*) and thymidine kinase (TK1/*orf51*) also clustered in Phase 1. Those two proteins (and DNA polymerase B – the same type of DNA polymerase encoded by nudiviruses) were all proposed to be involved in a pathway that oversees the misincorporation of uracil residues into viral DNA to ensure the fidelity of viral DNA genome replication [50]. Congruently, two genes that encode for a deoxynucleoside kinase (DNK/*orf71*) and thymidylate synthase (TS/*orf109*), both known to be involved in nucleotide metabolism [51,53], cluster in Phase 1. Furthermore, Phase 1 featured virus genes encoding for proteins with putative roles in host remodelling (i.e. inhibition apoptosis or host gene expression), including a variant of the inhibitor of apoptosis type 3 protein (IAP/*orf138*), a protein with a histone-like domain (histones H3 and H4/*orf1*), a histidine kinase (*orf3*) and a methyltransferase (MT/*orf37*). Almost half of the nudiviral core genes (12 in total) were assigned to Phase 2: *fen-1*, *helicase*, *helicase-2*, *integrase*, *pif-1*, *pif-5*, *GbNV_gp67-like*, *11K*, *p33*, *p6.9*, *vp39* and *vp91*. Accessory genes assigned to Phase 2 encode for a DNA ligase III (*orf36*), two thymidine kinases (TK2/*orf111* and TK3/*orf115*) and two ribonucleotide reductases (RR1/*orf95* and RR2/*orf73*). Other notable genes within Phase 2 include a dihydrofolate reductase (DHFR/*orf7*), a glycine hydroxymethyltransferase (GHMT/*orf72*)*,* and the virion structural protein 28K (*orf99*). Helicase, helicase-2, FEN-1, integrase and DNA ligase III have been labelled as proteins involved in DNA replication/processing [2], while orthologues of the proteins TK2 and TK3 [54], RR [55,56] and DHFR and GHMT are all associated with nucleotide metabolism [57], which includes processes essential for deoxynucleotide synthesis and thus DNA replication. The nudivirus core gene *GbNV_gp67-like* is thought to encode a microtubule-associated protein with a putative function in viral replication by remodelling the microtubule network of infected cells [58,59], but experimental data are needed to verify that this nudiviral protein acts in the same way as homologues in other lefavirals. The *pif-1* and *pif-5* genes encode proteins known as *per os* infectivity (PIF) factors (PIF-1 and PIF-5, respectively), which play an essential role in oral infectivity as structural components of the occlusion-derived virions (ODVs) in baculoviruses [60,61]. PIF homologues from bracoviruses, hytrosaviruses, nudiviruses and the more distantly related nimaviruses (*Nimaviridae*) are thought to serve similar functions [2,62]. Of the remaining genes mentioned above, some encode homologues of proteins known to be involved in nucleocapsid assembly (*vp39*, *vp91*), virion production and envelopment (*p33*) [63,67] or in viral DNA processing (*p6.9*) [2,68]. Both the core gene *11K* and the accessory gene *28K* encode virion structural proteins [69]. In summary, 58.82% of genes (20 out of 34) expressed in the early stages of HzNV-1 infection (first 6 h) are more or less directly associated with viral DNA replication and transcription, while 26.47% encode virion components. A significant increase in the relative level of viral DNA was measured from 7 hpi onwards (Fig. S4 in Supplementary Material 1), which logically confirms the observed timeline and the genes found to be expressed in Phase 2: first the expression of all genes necessary for the virus replication process, followed by the replication of the virus.

Genes that are abundantly expressed in Phase 3 are mostly nudiviral core genes related to infectivity (*p74*, *pif-2*, *pif-3* and *pif-4*), virion morphogenesis (*38* k, *ac81* and *GbNV_gp19-lik*e) or genes with unknown functions (*GbNV_gp58-lik*e). Phase 3 accessory genes include *orf100*, which encodes a ribonuclease H-like/RNase protein (RNHL), as well as *orf135*, which encodes a second inhibitor of apoptosis protein. The PIF-encoding genes (*pif-1* and *pif-5*), mentioned above in Phase 2, are still expressed in Phase 3. Homologues of all the mentioned-*pif* genes facilitate the fusion of baculoviral ODVs with host midgut cells [2,61], while homologues of the proteins 38K, AC81 and 15K ensure proper nucleocapsid assembly and envelopment [63,66, 70, 71]. While the function of the protein encoded by GbNV_gp19-like has not yet been experimentally characterized, it contains an α/β hydrolase domain, as identified by InterPro [72]. Further analysis using the HMMER web tool [73] classified GbNV_gp19-like within the carboxymethylbutenolide lactonase family, although this top match was supported by only a marginally significant *E*-value of 4.2E−03. Similar to *orf138* in Phase 2, the IAP protein encoded by *orf135* likely facilitates prolonged survival of host cells, providing the virus with additional time to replicate, a mechanism akin to that demonstrated for IAP homologues in baculoviruses [74].

In the latest stage, Phase 4, all the HzNV-1 genes are being expressed with the exception of the two core genes *pif-6* and *vlf-1*. All Phase 4 genes are expressed at low levels even at 24 hpi. In baculoviruses, PIF-6 cooperates with PIF-4 to stabilize the ODV entry core complex for better proteolytic resistance [75]. Previous studies demonstrated that the baculovirus homologue of the very late expression factor 1 (VLF-1) protein is a structural component of the nucleocapsid. It is hypothesized to execute the crucial final step of processing large concatemeric viral DNA intermediates into lengths suitable for proper packaging within the nucleocapsid [76], which is consistent with VLF-1 expression occurring during the latest stage of viral infection.

Based on our clustering approach, we can deduce that HzNV-1 genes with transcription- and replication-associated functions initialize viral DNA replication in the earlier infection stages (Phases 1 and 2), while viral genes are mostly involved in virion maturation and assembly showed expression at a more advanced stage of infection (Phases 3 and 4) (Fig. 2b). Coherent with what is known from baculovirus studies, virus replication and transcription are needed to establish late gene expression through the viral RNA polymerase, while early genes are usually transcribed by a host RNA polymerase [77,79]. Early expressed accessory genes, encoding proteins such as viral thymidine kinases (*tk1*, *tk2* and *tk3*), viral ribonucleotide reductases (*rr1* and *rr2*) or deoxynucleotide kinase (*orf71*), allow the virus to partially circumvent the metabolic limitations of its host by using this arsenal to aid nucleic acid production [80]. The expression of these genes at the onset of HzNV-1 infection likely contributes to the creation of optimal conditions for viral DNA synthesis and, hence, replication.

Regarding the expression kinetics of the nudiviral core genes, similar results were previously obtained for the endogenous nudivirus harboured by the wasp *Cotesia congregata* (Cotesia congregata bracovirus) [21]. Accordingly, transcription-associated genes (*p47*, *lef-9*, *lef-8*, *lef-4* and *lef-5*) were found to be more highly expressed than most other genes in the ovaries of the earliest developmental wasp stage, followed by genes involved in replication (*helicase* and *fen-1*), while structural nudiviral core genes reached their highest expression levels in the later developmental stages of the wasp. Thus, despite undergoing endogenization, the nudiviral core genes maintained in bracoviruses have largely retained the gene expression profiles of their free-living nudiviral ancestor.

### A putative HzNV-1 promoter motif regulates early gene expression primarily associated with viral genes for transcription and replication

**Fig. 3.**
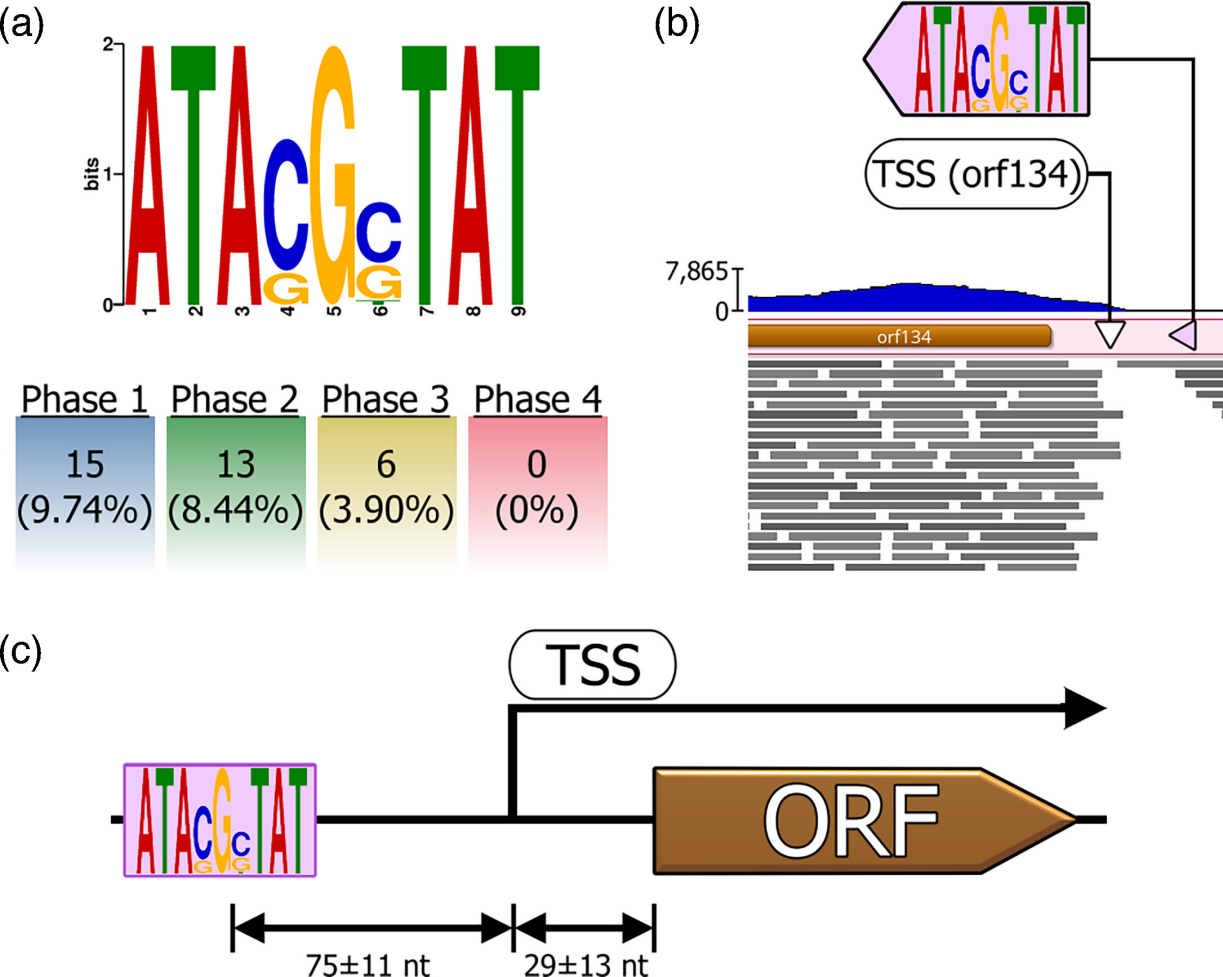
Analysis of the newly identified HzNV-1 motif, showing its prevalence and position relative to TSS upstream of HzNV-1 genes. (**a**) Sequence logo of the significantly enriched motif ‘ATA[G/C]G[G/C]TAT’ identified from the 300 nt upstream sequences of all HzNV-1 genes. The proportion of Phase 1, Phase 2, Phase 3 and Phase 4 genes among all 154 HzNV-1 genes with the motif are indicated as percentages in brackets. (**b**) Exemplary mapping of the paired-end RNA-seq reads (6 hpi) in the upstream region of HzNV-1′s orf134 along with the TSS and ‘ATA[G/C]G[G/C]TAT’ motif visualized with Geneious. The motif of orf134 is shown (purple triangles) to exemplify the distance of the motif and start codon to the TSS (white triangle) identified by TSSr. The read coverage graph is displayed as a blue curve with scale on the left and predicted ORF in brown. (**c**) Schematic showing the mean±sem nt distances between motif, start codon and TSS calculated from all ATA[G/C]G[G/C]TAT-containing HzNV-1 genes.

As part of the search for novel promoter sequences thought to be involved in early and late expression of the HzNV-1 genes, a comparison of the upstream sequences of all HzNV-1 genes was performed, with respect to the four identified phases. When the 300 bp upstream sequences of the genes assigned to Phases 1 to 3 were compared using MEME analysis, a motif corresponding to the consensus sequence ATA[G/C]G[G/C]TAT stood out significantly (*E*-value=9.1E−012; (Fig. 3a). With no allowed mismatches, it was identified in the upstream sequences of 34 genes (Sheet 1 in Supplementary Material 2), representing 22.08% of all HzNV-1 genes. If a motif was located in the upstream sequences of two adjacent genes, it was counted separately for each gene, resulting in 15 genes in Phase 1, 13 genes in Phase 2, 6 genes in Phase 3 and none in Phase 4. Hence, this motif does not seem to be linked to a single temporal class of genes; however, it is preferentially found in early expressed genes (Phases 1 and 2, more rarely Phase 3). Additionally, using AME, the enriched ATA[G/C]G[G/C]TAT motif was tested for enrichment against sequences from related nudiviruses, including CafNV, CsNV, FrpNV, OrNV and ToNV, as well as the more distantly related baculovirus AcMNPV. The analyses indicated that this motif is significantly enriched in the upstream sequences of HzNV-1 when compared to the other nudiviruses and AcMNPV, as evidenced by significantly low *P*-values (1.21E−10 to 7.74E−09), whereas AME showed no significant difference in the occurrence of this motif between HzNV-1 and HzNV-2 (*P*-value=0.496). This suggests that the motif is specific to the genus *Betanudivirus* and does not appear to be conserved in the other nudiviruses, which served as the comparative reference points in this analysis. The Tomtom-assisted search for binding site motifs with homology to the ATA[G/C]G[G/C]TAT motif resulted in multiple alignments with binding sites of the transcription factor family ‘sine oculis homeobox’ (SIX; top score *P*-value=8.97E−04). The family of SIX proteins is a group of transcription factors that fulfils developmental functions in a broad range of organisms, including both vertebrates and invertebrates, and the evolutionary lineage of this family extends back nearly 500 million years [81]. Although the *P*-value obtained is only weakly significant, members of the SIX family are known for their RNA polymerase II (RNAP II)-specific DNA-binding properties [82,83], which would align with early gene expression.

We further inspected the degree of proximity of the predicted TSS and the translational start codon to the ATA[G/C]G[G/C]TAT motif for the respective HzNV-1 genes. The TSSr tool was able to predict the TSS for 28 of the 34 ATA[G/C]G[G/C]TAT motif-containing virus genes. We used this information to visualize (Fig. 3b) and calculate the mean distances from those TSS to the motif of interest and the start codons of the respective genes (Fig. 3c). The nt distances between motif and TSS (mean=75, sem=±11) showed similar variability as the distance of the translational start codon to the TSS (mean=29, sem=±13). While these results offer initial insights on the spatial dynamics of the ATA[G/C]G[G/C]TAT motif to paired-end RNA-seq read coverages, experimental data using accurate sequencing techniques such as CAGE- and TSS-seq will be necessary to confirm its role as a novel HzNV-1 motif involved in transcription.

Several genes with this motif appear to be involved in virus transcription and replication, including *lef-5*, *dnapol*, *helicase-2*, *tk1*, *tk2*, *rr1*, *rr2*, dUTPase (*orf69*) and deoxynucleotide kinase (DNK). Those genes are all of critical importance for early HzNV-1 infection and essential to initiate late viral gene expression and establish an optimal environment with nucleic acid components for viral genome synthesis. It is particularly noteworthy that some of these viral genes containing that motif (*tk1*, *tk2*, *rr1*, *rr2* and *orf69*) have been described to be of eukaryotic host origin and as having been acquired by the virus through horizontal gene transfer [57]. In the context of the host, a FIMO-assisted search for the ATA[G/C]G[G/C]TAT motif (no mismatches allowed) in the upstream sequences of *H. zea* genes resulted in 251 hits, of which 170 corresponded to annotated *H. zea* genes. Among those, there were host genes with similar functions as those HzNV-1 genes with the ATA[G/C]G[G/C]TAT motif, including an RNA polymerase, two helicases, and seven zinc finger proteins (Sheet 5 in Supplementary Material 2). Although there is a possibility that HzNV-1 acquired these host genes – as it has been mentioned for *tk1*, *tk2*, *rr1*, *rr2* and *orf69* – these *H. zea* genes do not exhibit sequence homology with the corresponding HzNV-1 ORFs. This suggests that if these genes were indeed acquired from the host along with their regulatory motifs, they have greatly diverged within the virus genome beyond recognition. In addition to capturing host genes to expand the viral genomic arsenal, the acquisition of host genes together with their binding motifs may have helped to improve the binding efficiency of the host RNA polymerase II to HzNV-1 promoter regions to ensure proper expression of these critical genes.

### Numbers of host DEGs fluctuate over the course of HzNV-1 infection

**Fig. 4.**
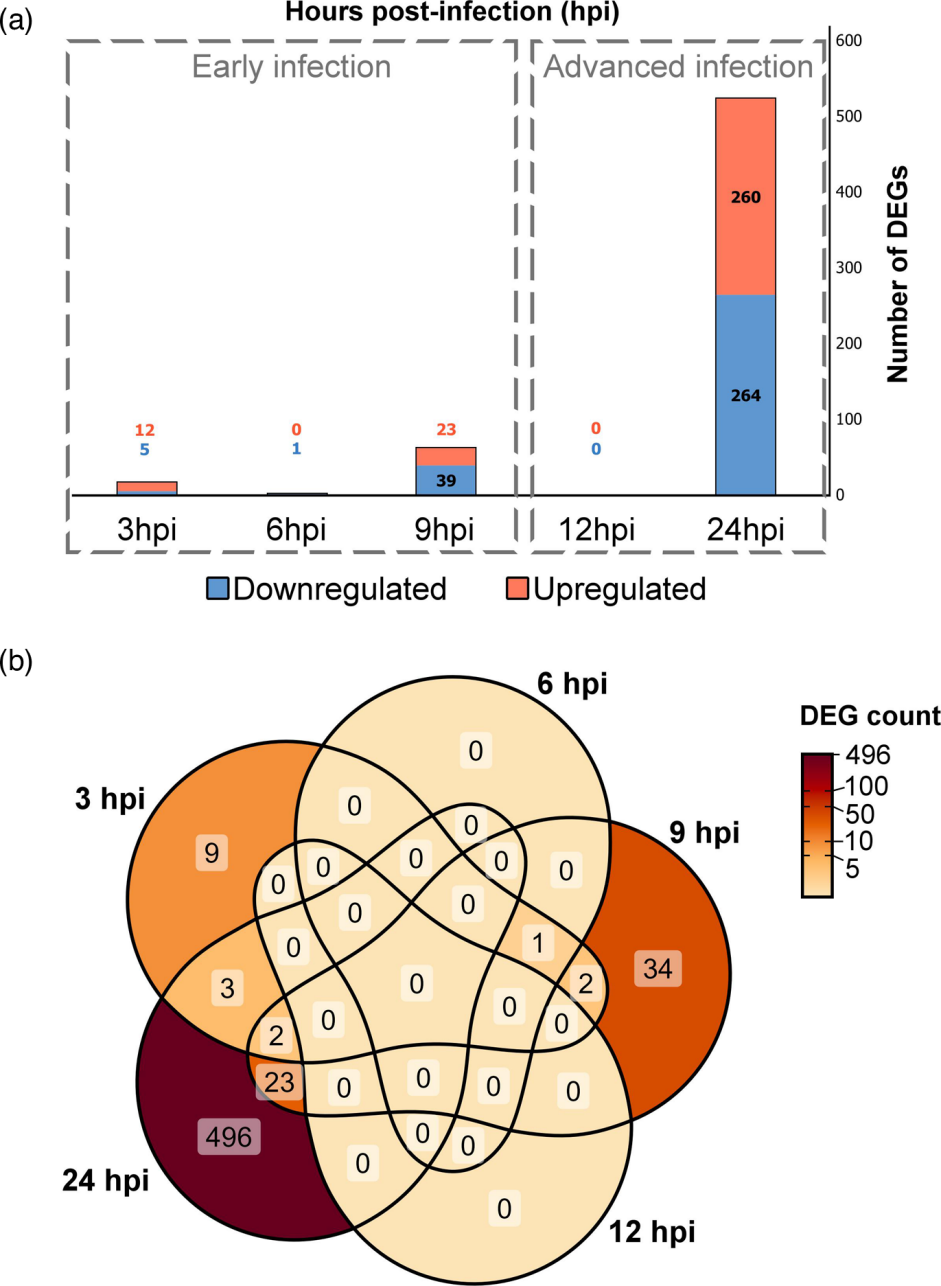
Overview of up- and downregulated host genes at different time points during HzNV-1 infection. (**a**) Changes in the number of up- (orange) and downregulated (blue) genes over the course of HzNV-1 infection summarized in a stacked column chart. (**b**) Venn diagram displaying the overlap of DEGs at 3, 6, 9, 12 and 24 hpi, showing distinct and shared gene expression profiles within and across the two individual experiments. The colour scale represents the number of (time-point overlapping) DEGs, with darker shades indicating a higher DEG count in the respective section.

Our analysis yielded a total of 570 distinct host DEGs, of which 74 were associated with the early stages of HzNV-1 infection (3, 6 and 9 hpi), and 524 DEGs were determined during advanced infection (12 and 24 hpi) when compared to mock-infected cells (Fig. 4a). Genes with an FDR value <0.05 and a log2(FC) >log2(1.5) were considered differentially expressed. The log2FC of all identified DEGs with their respective adjusted *P*-values can be found in the supplementary material (Sheet 2 in Supplementary Material 2). The time point-specific numbers of DEGs varied with progressing virus infection, including 12 up- and 5 downregulated genes at 3 hpi, 1 downregulated gene at 6 hpi, 23 up- and 39 downregulated genes at 9 hpi and no DEGs at 12 hpi. The number of DEGs reached its peak at 24 hpi with 260 up- and 264 downregulated genes, highlighting an overall greater amplitude of virus-induced transcriptional changes in the host cells during advanced infection. The ‘zigzag’-like fluctuations in the number of DEGs between 3 and 12 hpi resemble the well-known zigzag model in plant pathology, which depicts the interplay between host immune responses and viral counter-effectors over time [84]. Notably, like plants, insects also use RNA interference as an antiviral defence, and it is well documented that the RNA silencing pathway plays a key role in the zigzag model [85]. However, while the original zigzag model is qualitative and lacks specific units for measurement [86], our findings would suggest that the number of DEGs could serve as a quantifiable indicator of these fluctuating defence amplitudes during host–virus interactions. Naturally, additional experimental data would be needed to support this hypothesis. Moreover, any generalization to other biological systems should be approached with great caution.

A total of 28 particular DEGs were identified in both early and advanced stages of infection (Fig. 4b). Notably, none of the upregulated DEGs were shared across all three stages of early infection. However, four downregulated genes were common between the 3 and 9 hpi time points. Interestingly, the only DEG identified at 6 hpi was also detected as DEG at 3 and 9 hpi. This DEG encodes for an uncharacterized protein (LOC124637371) and, based on InterPro analysis, contains transmembrane helices and a cytoplasmic domain. This implies that the protein likely plays a role in interactions between the virus and host cellular membranes, potentially influencing viral replication or pathogenesis, especially during early infection. The majority of DEGs were unique to the advanced infection stage at 24 hpi, with only a few overlaps between early time points. The most significant overlap between early and advanced infection occurred between 9 and 24 hpi, with 5 upregulated and 18 downregulated genes shared between these stages. This highlights that certain host genes may play crucial roles in both early and advanced stages of nudivirus infection. Particularly, the decreased expression of the Sprouty (Spry) protein gene (LOC124632226) may facilitate HzNV-1 replication, similar to the findings for Bombyx mori nucleopolyhedrovirus, where its loss of function benefited the baculovirus and increased *Bombyx mori* mortality [87]. Three upregulated genes were found in common at both 3 and 24 hpi, including two major heat shock 70 kDa proteins Ba-like (LOC124642626 and LOC124642627) and a putative glutathione-specific gamma-glutamylcyclotransferase 2 (LOC124640872). These genes may play important roles in both early stress responses and later stages of infection. Additionally, two genes were significantly downregulated across three early time points (3, 9 and 24 hpi), encoding for phosphatidate phosphatase LPIN2 (LOC124644440) and interferon regulatory factor 2-binding protein 1 (LOC124636415), suggesting that their consistent suppression is crucial for infection progression.

The ratio between the number of different downregulated genes (283) and upregulated genes (287) only showed a slight difference over the course of global HzNV-1 infection. However, the log2FC of upregulated genes was of greater magnitude (highest measured log2FC=6.84) compared to those of downregulated genes (lowest measured log2FC=−1.83) (Table 1).

**Table 1.** Overview of the 15 most upregulated and 15 most downregulated DEGs with predicted protein functions considering all infection time points These DEGs are listed from the highest to the lowest FC value. Of these DEGs, some genes may be differently expressed at multiple time points. In this case, the time point at which they are more strongly regulated is taken as the reference and the second time point is marked with an asterisk (*).

NCBI gene symbol	Predicted function	Time point	log2FC	Adjusted *P*-value
**Upregulated genes**				
** LOC124642626**	Major heat shock 70 kDa protein Ba-like	24 hpi3 hpi*	6.842.29*	2.10E−113.32E−02*
** LOC124642627**	Major heat shock 70 kDa protein Ba-like	24 hpi3 hpi*	6.652.43*	1.33E−113.33E−02*
** LOC124630527**	Zinc finger protein 723-like	24 hpi	6.61	2.69E−04
** LOC124643760**	Extracellular serine/threonine protein kinase four-jointed	9 hpi	6.25	1.61E−02
** LOC124642339**	F-box/LRR-repeat protein 14	9 hpi	5.47	1.50E−02
** LOC124642779**	Lethal(2)essential for life-like (HSP)	24 hpi	4.65	3.95E−07
** LOC124642484**	Lethal(2)essential for life-like (HSP)	24 hpi	4.57	1.53E−06
** LOC124630722**	Heat shock protein 68-like	24 hpi	3.29	1.83E−12
** LOC124633640**	Lethal(2)essential for life-like (HSP)	24 hpi	3.29	2.13E−13
** LOC124642759**	Lethal(2)essential for life-like (HSP)	24 hpi	2.90	6.02E−12
** LOC124645234**	Zinc finger protein 260-like	9 hpi	2.64	2.16E−02
** LOC124642777**	Lethal(2)essential for life-like (HSP)	24 hpi	2.62	6.09E−10
** LOC124634467**	Lachesin-like	24 hpi	2.52	4.10E−05
** LOC124642780**	Lethal(2)essential for life-like (HSP)	24 hpi	2.26	1.39E−10
** LOC124640580**	Fibroblast growth factor receptor homologue 1-like	9 hpi	2.05	2.40E−02
**Downregulated genes**				
** LOC124629602**	Phosphoinositide 3-kinase adapter protein 1	24 hpi	−1.23	4.52E−05
** LOC124645702**	Laminin subunit alpha-2-like	9 hpi	−1.24	2.86E−02
** LOC124643685**	E-26-specific DNA-binding protein *pokkuri*	24 hpi	−1.26	5.88E−08
** LOC124644179**	Histone H1-like	9 hpi	−1.30	2.18E−02
** LOC124644180**	Histone H1-like	9 hpi	−1.31	2.13E−02
** LOC124645602**	Histone H1-like	9 hpi	−1.34	2.41E−02
** LOC124645603**	Histone H1-like	9 hpi	−1.36	2.21E−02
** LOC124645606**	Histone H2A	9 hpi	−1.38	2.21E−02
** LOC124632937**	Mid1-interacting protein 1A	9 hpi	−1.44	3.27E−02
** LOC124638833**	Probable ribonuclease ZC3H12B	9 hpi	−1.45	2.97E−02
** LOC124637172**	Protein giant-lens	9 hpi	−1.58	1.50E−02
** LOC124645608**	Histone H4	9 hpi	−1.60	4.90E−02
** LOC124645605**	Histone H3	9 hpi	−1.63	2.21E−02
** LOC124633107**	Ecdysone oxidase-like	24 hpi	−1.71	4.67E−02
** LOC124632048**	Von Willebrand factor D and EGF domain-containing protein	9 hpi3 hpi*	−1.72−1.35	1.50E−023.32E−02

Notably, among the most upregulated DEGs with available protein annotations (Table 1), four genes were identified early in infection, i.e. 9 hpi. These early DEGs encode an extracellular serine/threonine protein kinase, an F-box/LRR-repeat protein, a zinc finger protein and a fibroblast growth factor receptor. By 24 hpi, the remaining most upregulated DEGs predominantly encode several heat shock proteins (HSPs) and an additional zinc finger protein. The most downregulated DEGs during viral infection were detected primarily at 9 hpi and included genes encoding laminin subunit alpha-2-like, most histone proteins (H1, H2A, H3 and H4), mid1-interacting protein 1A, a probable ribonuclease, protein giant-lens and a protein containing the von Willebrand factor D and an EGF domain. Three more downregulated DEGs encoding a phosphoinositide 3-kinase adapter protein, E-26-specific DNA-binding protein (*pokkuri*) and an ecdysone oxidase were identified by 24 hpi. To gain deeper insights into these host gene expression changes, the next section will explore the functional grouping and potential interactions of all identified DEGs, underscoring their importance in cellular pathways and molecular processes and suggesting biological functions impacted by the HzNV-1 infection.

### Expression of host genes associated with cellular and metabolic pathways is modulated upon HzNV-1 infection

**Fig. 5.**
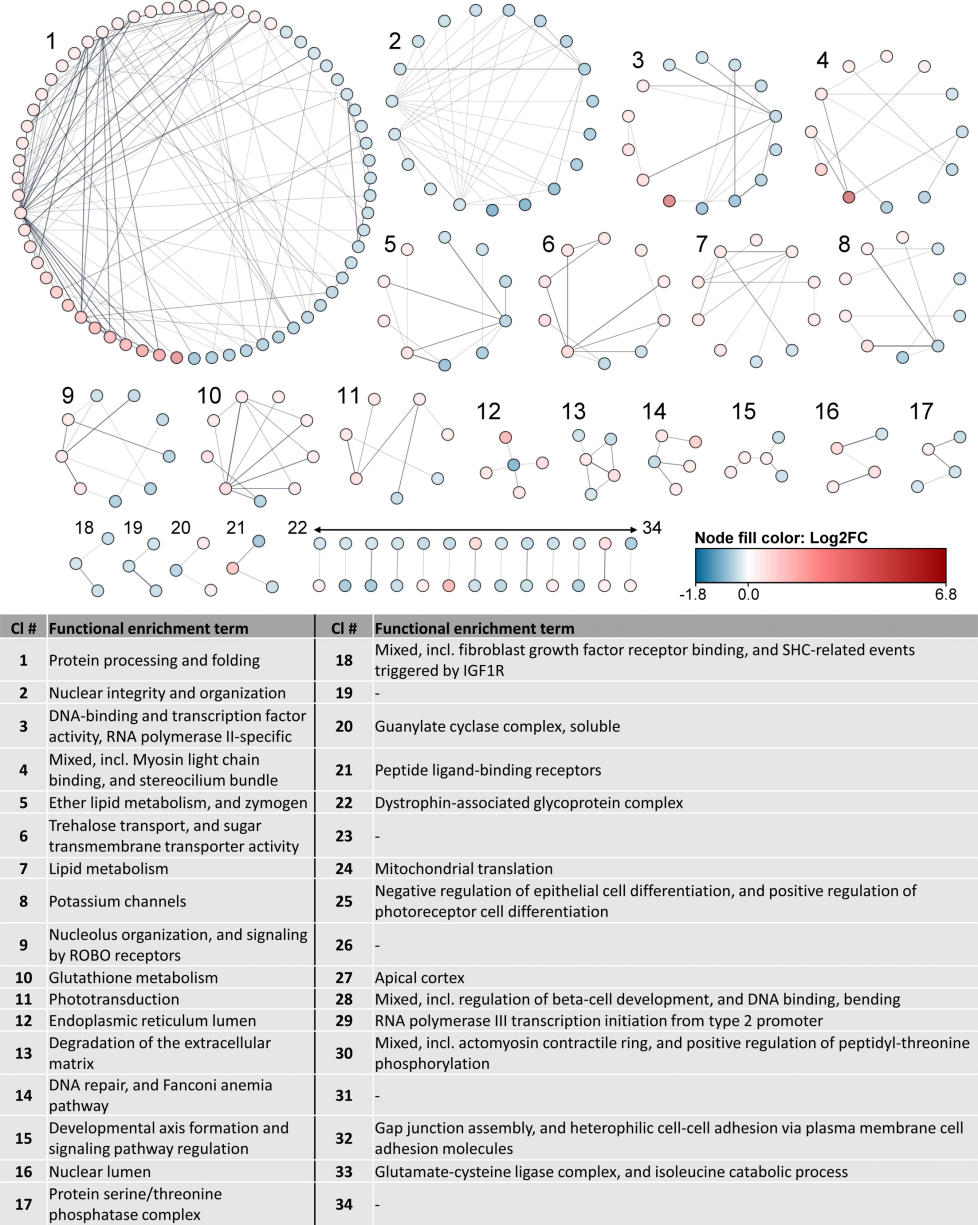
Clustered protein-protein interaction networks with functional enrichment terms derived from host DEGs during HzNV-1 infection. MCL clustering produced 34 distinct clusters, representing 239 connected proteins, with clusters numbered by size. Node colours reflect the log2FC of each DEG, ranging from downregulated (blue) to upregulated (red), as indicated by the colour bar. The thickness of grey edges between nodes corresponds to the strength of the interaction evidence. Functional enrichment terms for each cluster are listed in the accompanying table. Detailed *P*-values for each cluster and precise log2FC values for individual DEGs are available in Supplementary Material 2, Sheet 4.

All the differentially expressed host genes upon HzNV-1 challenge were subjected to a functional enrichment analysis using the web-based STRING database. Database-available interactions among DEGs were determined with a significant protein–protein interaction (PPI) enrichment *P*-value of 0.0232 for the global PPI network (Fig. S5A in Supplementary Material 1) . Based on the global STRING network, the most significant Gene Ontology, Kyoto Encyclopedia of Genes and Genomes and Local Network Cluster terms relate to protein processing and folding and nuclear integrity. Other enriched terms relate to the transcriptional machinery and DNA damage response (DDR) (Fig. S5B in Supplementary Material 1. The MCL-based clustering assigned 239 of the 570 DEGs (41.93%) to clusters with direct protein interactions; however, enriched functionality terms were unavailable for a number of DEGs because the *H. zea* proteome comprises numerous proteins without functional characterization. The proteins with akin functionalities and direct interactions were grouped into 34 clusters, and an overview from the individually extracted cluster networks was created (Fig. 5).

The inferred 34 clusters ranged in size from 63 to 2 DEGs, with cluster 1 and cluster 2 containing the most DEGs of all clusters. Cluster 1 is the largest cluster and comprises DEGs involved in protein processing and folding with 57.14% of DEGs being upregulated (red) and 42.86% being downregulated (blue). Cluster 2 has solely downregulated DEGs whose encoded proteins relate to nuclear and genome integrity, including structural constituents of chromatin, nucleosome assembly and organization and DDR. The two largest protein clusters will be thoroughly discussed in the two upcoming sections. The third largest cluster (cluster 3) comprised proteins related to the term ‘DNA-binding and transcription factor activity, RNA polymerase II-specific’, which implies that HzNV-1 infection modulates the transcriptional machinery of the host. Similar findings have been reported for the African swine fever virus, which promotes the expression of its genes by compartmentalizing viral mRNA, ribosomes and cellular translation factors within the virus factory [88]. The proteins of the fourth largest cluster (cluster 4) were enriched under the functional term ‘myosin binding and stereocilium bundle’. The differential expression of genes encoding for myosin- and stereocilium-related proteins during HzNV-1 is intriguing, since those proteins ensure proper stability of the cytosekeleton [89]. Congruently, multiple studies have shown that baculoviruses manipulate host cytoskeletal components to promote their intracellular trafficking and virion assembly [90,92], so it is possible that nudiviruses and baculoviruses share this pathological mechanism. Additionally, similar to what has been observed for baculoviruses [93,94], metabolic pathways such as glucose, lipid and amino acid metabolism were affected by HzNV-1 infection (clusters 5, 6, 7 and 10). Regulating and hijacking host metabolite synthesis is a common viral strategy, ensuring the virus has access to a sufficient pool of resources necessary for efficient propagation and replication [95,97].

### Host protein folding and processing machinery are greatly affected during advanced HzNV-1 infection

The analysis of protein–protein association networks revealed the largest cluster of DEGs (cluster 1) encoding for proteins involved in protein processing and folding, including various HSPs. Two HSPs (LOC124642627 and LOC124642626) from the HSP70 family showed significant upregulation at 3 and 24 hpi, with log2FCs of 6.65 and 6.84, respectively. Heat shock proteins, such as HSP70, function as molecular chaperones that fold various proteins or may play a role in DNA repair [98], and therefore, increased HSP-coding gene expression is a primary defence against stressors, including pathogen infections [99,100]. For instance, *Drosophila melanogaster* infected with *Drosophila* C virus (*Dicistroviridae*) exhibited an increased heat shock response to limit infection, while the loss of an essential heat shock transcription factor made flies hypersensitive to viral infection [101]. During infection of Sf9 cells with the baculovirus AcMNPV or of *Litopenaeus vannamei* shrimp with the white spot syndrome virus (WSSV; *Nimaviridae*), upregulation of HSP70 gene homologues was also observed [102,103]. Other upregulated DEGs associated with the heat shock response at 24 hpi in our study included genes encoding HSP 68-like, 97 kDa HSP, HSP-12.2-like, HSP 83 and activator of 90 kDa HSP ATPase homologue 1. In addition, genes encoding several members of the DnaJ/HSP40 family, such as DnaJA1, DnaJC10 and Hdj1, also showed increased expression. Overexpression of genes encoding Hdj1 can inhibit hepatitis B virus replication in humans, while DnaJA1 and DnaJC10/ERdj5 can benefit certain viruses, enhancing viral activities [104,107]. In general, it is a recurrent characteristic of viruses to hijack the host’s chaperone machinery to promote their replication [108,109].

Additionally, a total of six variants of the lethal(2)-essential-for-life [*l(2)efl*] gene encoding putative members of the HSP20 family were significantly upregulated at 24 hpi, with log2FCs ranging from 4.79 to 25.10. In *Drosophila*, these genes have been shown to be essential for viability, and their deficiency increases mortality [110,111]. The expression of *l(2)efl* can be induced by various stressors, including virus infections. This gene was identified as a shared DEG in honey bee batches infected with a number of viruses [112] and was upregulated during Nora virus infection in *D. melanogaster* [113]. Overexpression of *l(2)efl* in *Aedes aegypti* inhibited Dengue virus replication, while silencing stimulated virus replication [114].

In summary, the largest cluster of HzNV-1 infection-induced DEGs relates to the heat shock response and the protein folding machinery, highlighting the importance of this protein cluster in the innate immune response against insect virus infections and its susceptibility to hijacking by viruses [109,115, 116].

### HzNV-1 alters the expression of host genes involved in chromatin and nuclear dynamics during early and advanced infection

During HzNV-1 infection, the expression of genes encoding histone proteins was significantly downregulated (cluster 2). Histone H1 variants and histone H3 showed reduced expression at both 9 and 24 hpi (Sheet 2 in Supplementary Material 2). Histone H2A and H4 encoding genes were downregulated at 9 hpi, while histone H2B encoding genes were downregulated at 24 hpi. Hence, the gene regulation for histones H2A, H3 and H4, which form the nucleosomal core, and linker-histone H1 [117], were all negatively affected by HzNV-1 infection.

In eukaryotes, histones are crucial for DNA condensation into chromatin, for transcription and replication [118], and histones can fulfil roles in cell signalling, innate immunity and antimicrobial activities [117,119, 120]. Similarly, other members of the *Naldaviricetes (Lefavirales+Nimaviridae*) have been shown to downregulate histone gene expression, presumably to disrupt host transcription and facilitate virus replication. For instance, WSSV and Spodoptera litura nucleopolyhedrovirus (SpltNPV) infections were shown to reduce core histone levels (transcriptomic level, WSSV; proteomic level, SpltNPV) in shrimp and Sf21 cells, respectively [121,122]. Moreover, AcMNPV infection decreased levels of transcripts encoding for H2A, H3 and H4 in Sf cells [123]. Our findings further revealed downregulation of two genes encoding for histone-lysine methyltransferases (LOC124638756 and LOC124641771) and the gene of the zinc finger protein Gfi-1 (LOC124646193). Gfi-1 is a conserved transcriptional repressor whose deletion in mice results in decreased histone H2B levels [124,125]. Although little is known about the function of such enzymes in insects, the gene function might be similar. Thus, the downregulation of host histone-encoding genes during an HzNV-1 challenge is consistent with observations made for other naldaviruses.

**Fig. 6.**
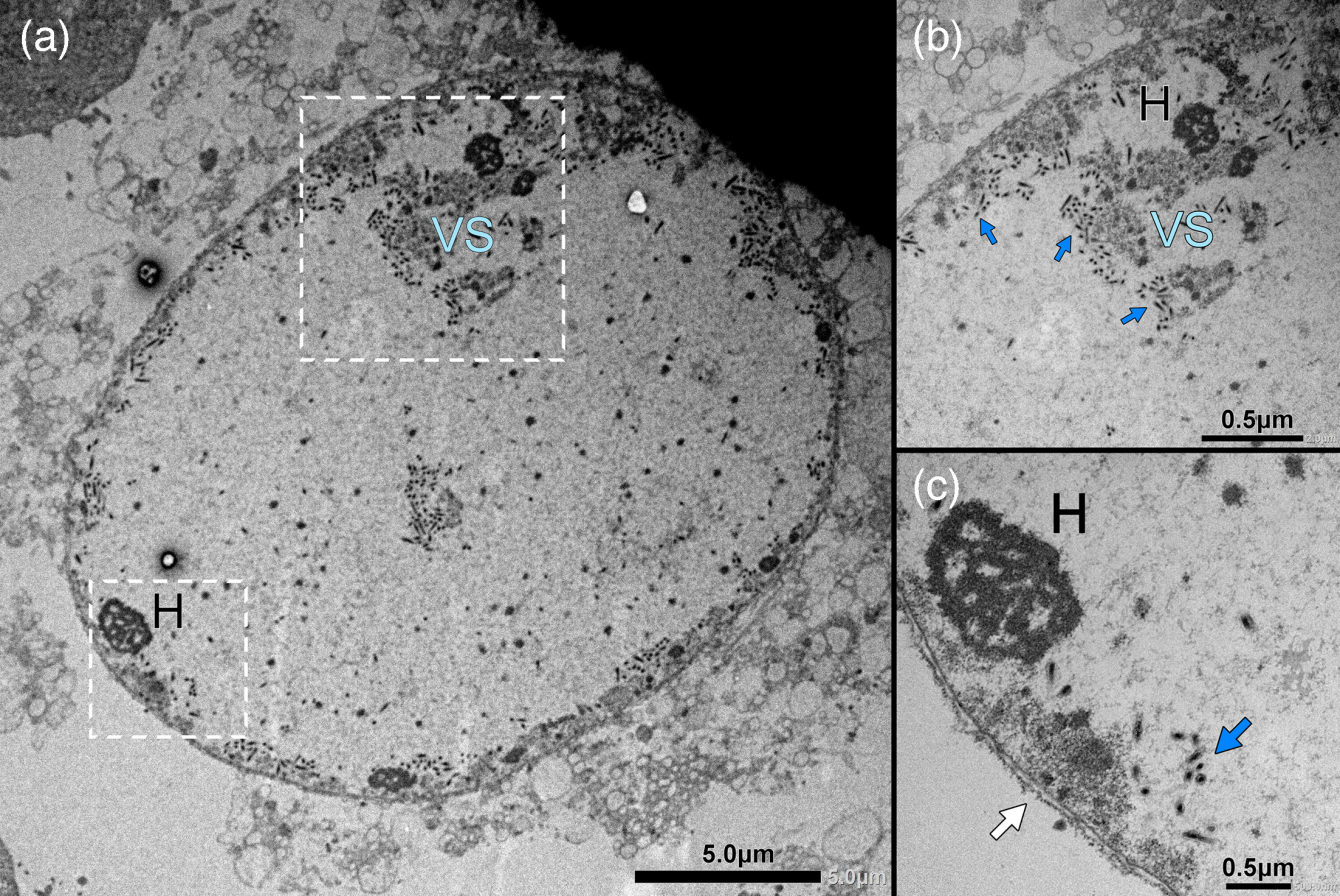
EM image of sectioned HZ-AM1 cell infected with HzNV-1 after 60 h of infection. (**a**) Whole view of an infected HZ-AM1 cell with indistinguishable nucleus and cytoplasm as well as electron-dense host chromatin (**h**). Bar, 5 µm. (**b**) Magnified region inside the right dashed box. Clusters of intracellular HzNV-1 virions (blue arrows) in proximity to the less-electron-dense virogenic stroma (VS). Bar, 0.5 µm. (**c**) Magnified region inside the left dashed box. Extracellular host cell membrane (white arrow) with HzNV-1 virions (blue arrows). Bar, 0.5 µm. EM images courtesy Jan W. M. van Lent, Wageningen Electron Microscopy Centre (WEMC).

Nevertheless, the differential expression of genes encoding proteins associated with nuclear integrity and organization indicates that nudivirus-induced processes may influence host chromatin dynamics and DNA packaging or even degrade the host cell nuclear lamina. The latter would align with observations made in granuloviruses (*Baculoviridae*) [126,127]. Chromatin, a DNA-protein complex condensed by histones [128], is essential for maintaining the integrity of the nuclear membrane through its interaction with lamins [129]. Consequently, the virus-induced downregulation of histone-encoding genes may severely impair the structural organization of the nuclear lamina, eventually leading to nuclear disintegration. The expected consequence of such a process is clearly visualized in electron microscopy (EM) images of an HzNV-1-infected HZ-AM1 cell at 60 hpi, showing that the nucleus and cytoplasm of the cell are no longer distinguishable from one another (Fig. 6).

The disintegration of the host nuclear membrane by viruses with intranuclear DNA replication has been documented in multiple studies [130,134], but it has not yet been observed for a nudivirus, except in the case of bracovirus particle release [135,136]. Our findings support the occurrence of this cytopathological process during HzNV-1 infection, similar to observations for related granuloviruses (*Baculoviridae*).

In this context, molecular mimicry is a common strategy used by viruses to interfere with host-specific cellular functions [137]. Notably, lepidopteran nudiviruses are currently the only known exogenous nudiviruses whose genomes encode a viral ‘histone mimic’ protein (ORF1) consisting of a long N-terminal tail and two histone-fold domains (InterPro ID: IPR009072). Although the role of this viral histone is not fully understood, it might play a role in disrupting the integrity of the host nucleus. Such histone mimics have been identified in the evolutionary-related bracoviruses with functions in suppressing host immunity [138,139]. In more distantly related viruses, histone mimics have also been shown to fulfil functions in host immune suppression, as well as in viral genome condensation, and interaction with the host DDR [140,141]. However, functional studies are required to determine whether the histone mimic encoded by *orf1* can play similar roles for HzNV-1.

## Conclusions

Monitoring the transcriptional changes in HZ-AM1 cells infected with HzNV-1 across five time points (3, 6, 9, 12 and 24 hpi) provided comprehensive gene expression profiles for both the virus and its host. Based on our study, we infer that the tipping point at which HzNV-1 finally takes over its host’s cellular machinery and reprograms the host cell into a viral factory occurs somewhere between 12 and 24 hpi. This observation is based on the drastic increase in detected DEGs at 24 hpi compared to the number of DEGs at the previously measured time points. Next to the total number of DEGs, we observed ‘zigzag’-like fluctuations in the quantities of DEGs between 3 and 12 hpi, suggesting that the number of DEGs might be a quantifiable indicator of changing defence amplitudes during insect–virus interactions.

We clustered the 154 HzNV-1 genes into four temporal classes, showing that genes linked to virus transcription and replication mostly associated with the two earliest phases (Phases 1 and 2) of infection, while the phases of advanced infection (Phases 3 and 4) mostly harboured genes involved in virion assembly and maturation. Additionally, we identified a putative new promoter motif in the genome of HzNV-1 that predominantly associated with early expressed genes, mainly involved in transcription and replication. During HzNV-1 infection, 570 DEGs of the host were identified, with notable association to protein processing, nuclear and cytoskeleton integrity, as well as metabolic pathways associated with glucose, lipid and amino acid metabolism. Significant upregulation of certain heat shock proteins and downregulation of histones were observed, indicating disruption of the host cellular machinery to facilitate viral replication and impair host defence mechanisms.

The lepidopteran betanudivirus, HzNV-1, is a particular example of a nudivirus, given its cell culture-restricted pathogenicity, possession of a histone-like protein, tropism for reproductive organs and extraordinary genome dimensions compared to other members of the *Nudiviridae*. Hence, comparisons of our results to nudiviruses of the other genera should be carefully contemplated. Our *in vitro* system does provide a detailed examination of HzNV-1 pathogenesis in an ovarian cell-derived cell line of *H. zea* but is also limited when compared to *in vivo* studies with HzNV-2. On the other hand, the cell line infection allowed for a much more synchronized infection, facilitating the discrimination of particular phases in the viral invasion (Fig. 2).

## supplementary material

10.1099/jgv.0.002066 Supplementary Material 1.

10.1099/jgv.0.002066 Supplementary Material 2.

## Abbreviations

AcMNPV: Autographa californica multiple nucleopolyhydrovirus
AME: Analysis of Motif Enrichment
DDR: DNA damage response
DEGs: differentially expressed genes
DHFR: dihydrofolate reductase
EM: electron microscopy
FDR: false discovery rate
FIMO: Find Individual Motif Occurrences
GHMT: glycine hydroxymethyltransferase
hpi: hours post-infection
HSPs: heat shock proteins
HzNV-1: Heliothis zea nudivirus 1
HzNV-2: Helicoverpa zea nudivirus 2
log2FC: log2 fold change
MCL: Markov Cluster Algorithm
MOI: multiplicity of infection
ODVs: occlusion-derived virions
OrNV: Oryctes rhinoceros nudivirus
PIF: *per os* infectivity
PPI: protein–protein interaction
SIX: sine oculis homeobox
SpltNPV: Spodoptera litura nucleopolyhedrovirus
TCs: TSS clusters
TSS: transcription start sites
VLF-1: very late expression factor 1
WSSV: white spot syndrome virus
