# Supplementary material for: Transcriptional dynamics during Heliothis zea nudivirus 1 infection in an ovarian cell line from Helicoverpa zea
Source: J Gen Virol. 2025 Jan 13;106(1):002066. doi: 10.1099/jgv.0.002066 (PMC11728702; doi:10.1099/jgv.0.002066)
Supplement: Supplementary Material 1. [file jgv-106-02066-s001.pdf]

Visualization of differently expressed genes (DEGs) with volcano pots

Volcano plots showing the time point-specific distribution of up- and downregulated host genes during virus infection were inferred using the R package Glimma v2.4.0 (2).

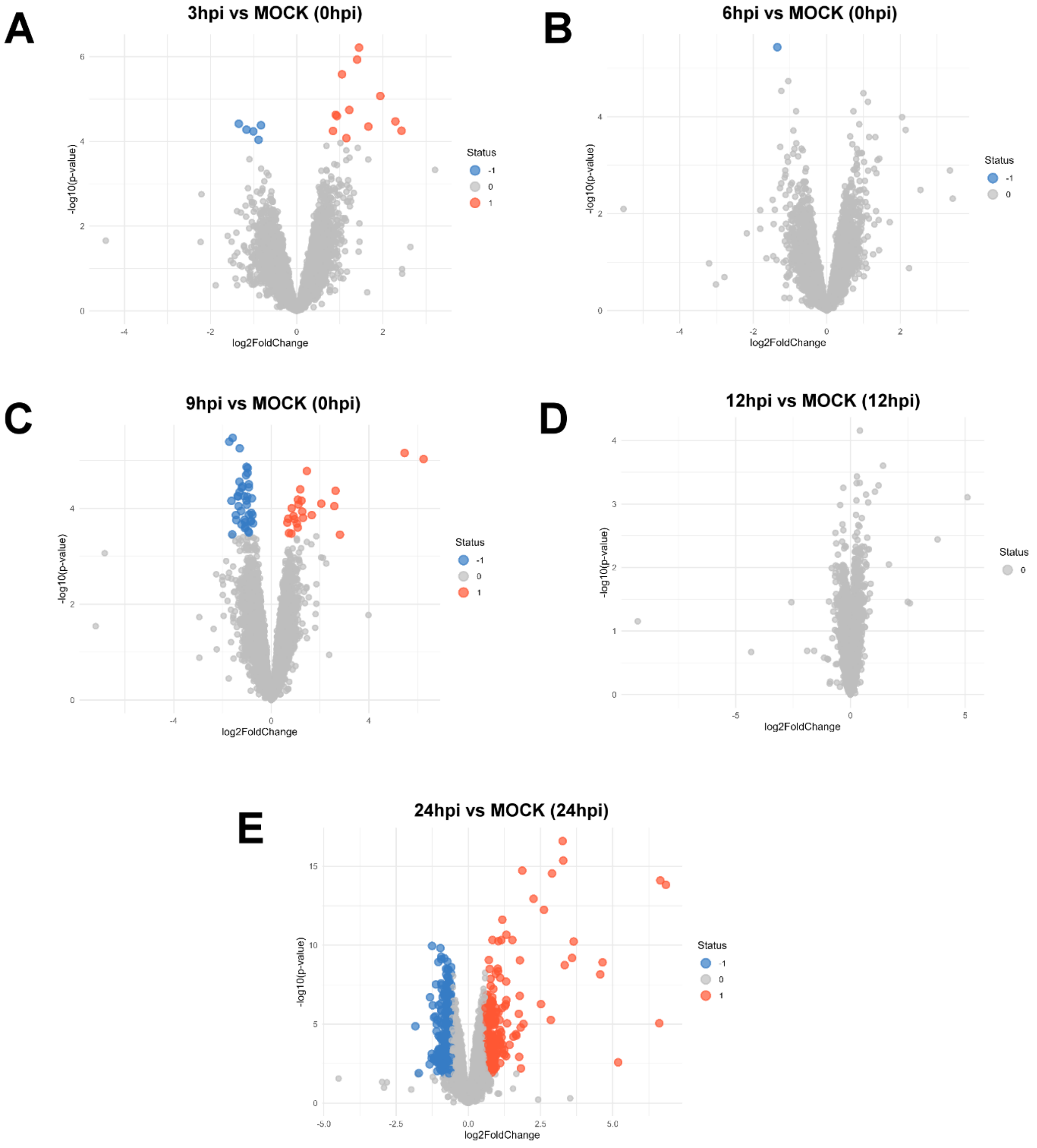

Fig. S1. (**A – C**) Volcano plots showing the time point-specific distributions of up- (orange dots) and downregulated (blue dots) host genes, and their expression changes at 3, 6 and 9 hpi in comparison to mock-infected cells (0 hpi). (**D – E**) Volcano plots showing the distributions of up- and downregulated host genes in infected cells at 12 hpi and 24 hpi, and their expression changes in comparison to mock-infected cells (12 hpi or 24 hpi, respectively). In the volcano plots, the y-axis shows the negative log10(p-value), while the x-axis displays the log2-fold changes (log2FC). The differing significance cutoffs in the volcano plots result from limma's

procedure to assign higher p-value cutoffs to contrasts with many DEGs, and lower p-value cutoffs to contrasts with fewer DEGs.

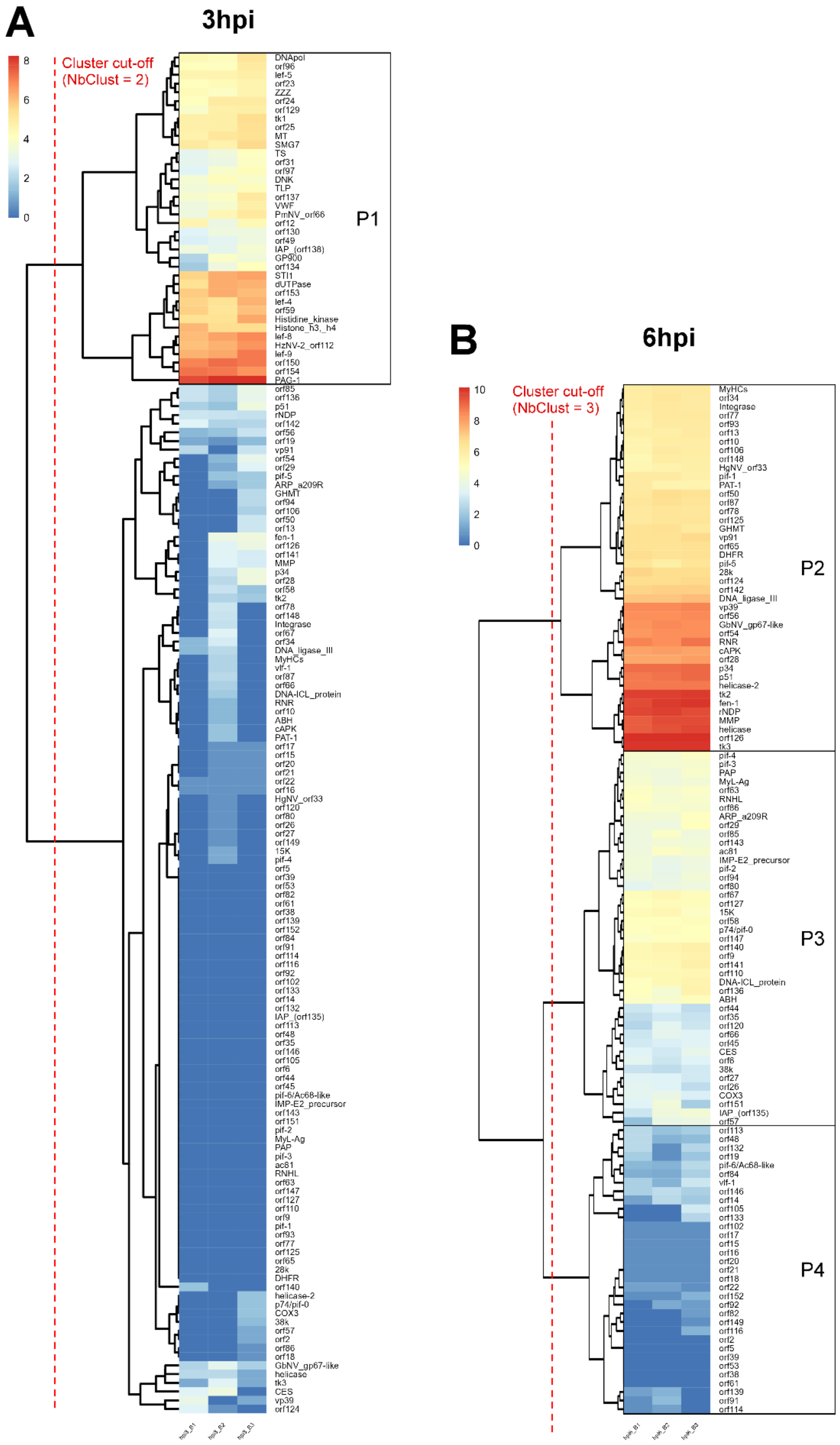

Fig. S2. Hierarchical clustering of normalized HzNV-1 gene counts from infection time points **(A)** 3 hpi and **(B)** 6 hpi heatmaps showing the expression levels of the HzNV-1 genes in the two earliest stages of infection. The optimal numbers of clusters (red dotted line) for both individual heatmaps, and the temporal classes of genes were accordingly assigned.

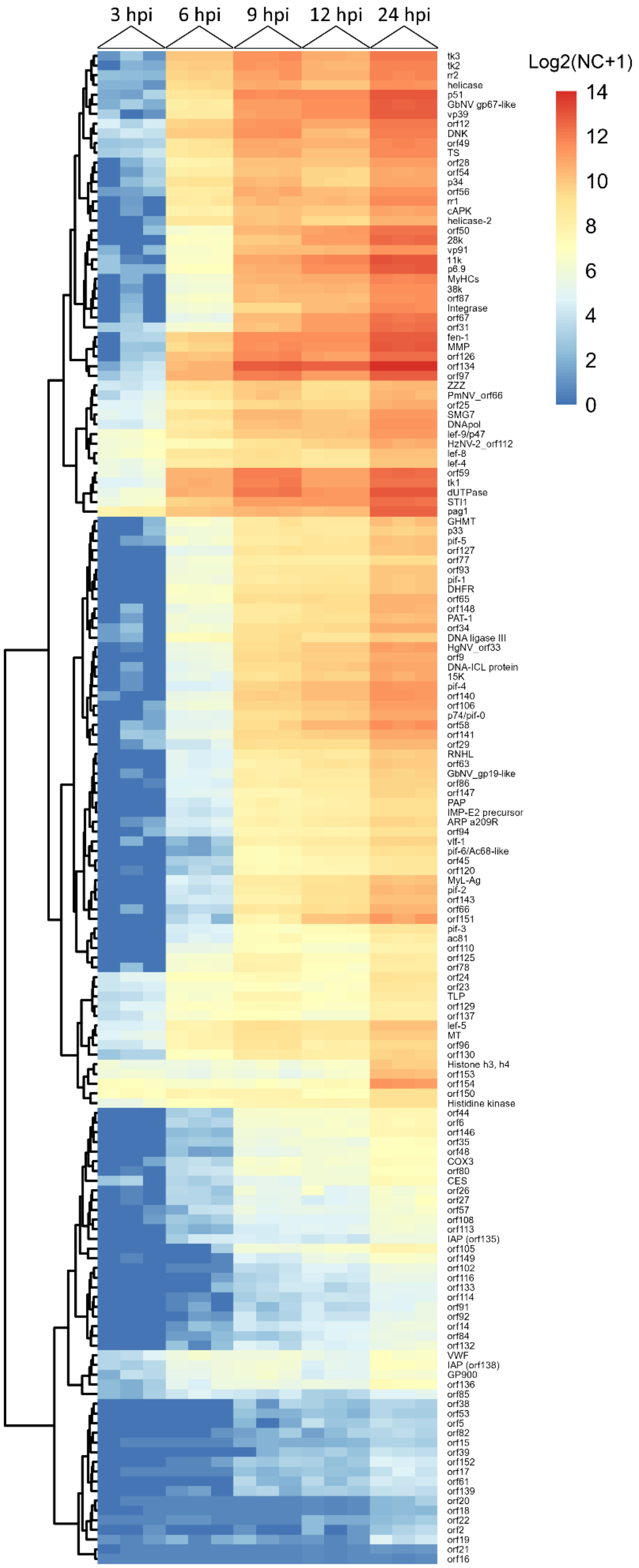

Fig. S3. Unsupervised hierarchical clustering of normalized HzNV-1 gene counts with data from all five time points as input. The dendrogram indicates clusters of genes that exhibit similar global expression patterns across the time course.

Table S1 Short reads information of each sample with percentages of respective Hisat2 alignments to the host (*Helicoverpa zea*) or virus (Heliothis zea nudivirus 1) genome. The percentages of reads mapped to the host (*H. zea*) and the virus (HzNV-1) represent the proportion of reads that aligned to each genome, relative to the total number of reads after trimming. I = virus-infected, C = mock-infected

Sample	Cond.	No. of reads	No. of reads after trim	No. of mapped reads to *H. zea* genome	Reads aligning to *H. zea* genome (%)	No. of unmapped reads after alignment to *H. zea* genome	No. of unmapped reads aligning to HzNV-1 genome	Unmapped reads aligning to HzNV-1 genome (%)
**0hpi_B1**	C	59,524,704	58,269,522	53,438,512	91.709	4,831,010	495	0.001
**0hpi_B2**	C	59,347,242	58,260,440	52,934,136	90.858	5,326,304	889	0.002
**0hpi_B3**	C	56,381,136	55,223,040	50,000,277	90.542	5,222,763	723	0.001
**3hpi_B1**	I	58,093,300	54,919,506	48,372,612	88.079	6,546,894	16,453	0.030
**3hpi_B2**	I	56,188,186	54,821,348	49,576,424	90.433	5,244,924	19,973	0.036
**3hpi_B3**	I	54,225,532	51,967,510	46,065,199	88.642	5,902,311	21,743	0.042
**6hpi_B1**	I	51,540,092	50,249,346	44,686,300	88.929	5,563,046	592,350	1.179
**6hpi_B2**	I	56,715,806	54,677,330	47,644,315	87.137	7,033,015	700,530	1.281
**6hpi_B3**	I	59,405,502	58,072,560	51,573,070	88.808	6,499,490	772,297	1.330
**9hpi_B1**	I	54,594,030	53,009,374	43,128,955	81.361	9,880,419	4,576,232	8.633
**9hpi_B2**	I	57,145,186	55,945,306	46,294,618	82.750	9,650,688	4,279,422	7.649
**9hpi_B3**	I	58,199,552	56,793,144	46,388,701	81.680	10,404,443	5,163,680	9.092
**12hpi_B1**	C	39,392,600	38,172,802	34,416,059	90.159	3,756,743	415	0.001
**12hpi_B2**	C	41,923,364	40,627,852	36,358,437	89.491	4,269,415	387	0.001
**12hpi_B3**	C	40,808,366	39,583,558	35,808,525	90.463	3,775,033	370	0.001
**12hpi_B1**	I	43,416,158	41,498,866	34,446,330	83.005	7,052,536	2,579,886	6.217
**12hpi_B2**	I	52,134,144	50,433,336	41,937,868	83.155	8,495,468	3,185,963	6.317
**12hpi_B3**	I	40,473,120	38,940,882	32,211,087	82.718	6,729,795	2,439,157	6.264
**24hpi_B1**	C	73,008,924	71,611,450	64,547,053	90.135	7,064,397	917	0.001
**24hpi_B2**	C	48,128,340	47,468,798	42,919,072	90.415	4,549,726	567	0.001
**24hpi_B3**	C	39,487,824	38,502,658	34,974,237	90.836	3,528,421	445	0.001
**24hpi_B1**	I	40,951,296	39,525,334	27,118,719	68.611	12,406,615	9,003,317	22.779
**24hpi_B2**	I	52,434,608	51,439,572	36,356,744	70.679	15,082,828	11,469,085	22.296
**24hpi_B3**	I	43,149,140	41,752670	28,471,398	68.191	13,281,272	9,537,254	22.842

Timepoint determination of HzNV-1 DNA replication initiation via quantitative PCR

Prior to conducting quantitative PCR (qPCR) analysis, two primer pairs were designed using SnapGene software (www.snapgene.com), with one set targeting the host and the other targeting the virus. Host-specific primers were designed to amplify a 199-bp fragment from the gene encoding Glyceraldehyde 3-phosphate dehydrogenase (GAPDH). The sequences for the host-specific primers were: qPCR_GAPDH_fw: 5'-GGTGGTGCTAAGAAGGTCATCATC-3' and qPCR_GAPDH_rev: 5'-CGTGTACAGTGGTCATCAGACC-3'. For the virus, primers were designed to bind in the coding region of the *vlf-1* gene, producing a 155-bp amplicon. The sequences for the virus-specific primers were: qPCR_vlf-1_fw: 5'-GTTCAAGGTGGAGGTCGACTC-3' and qPCR_vlf-1_rev: 5'-GCAAGCGATCCTGATTCTGTTC-3'. Both primer sets were designed with an annealing temperature of 59°C. The amplification efficiency of each primer pair was determined using a serial dilution of DNA extracted from HZNV-1-infected HZ-AM1 cells. The Cq values were

measured in technical triplicates using the Bio-Rad CFX96 Touch Real-Time PCR system. The slope of the standard curve derived from these Cq values was used to calculate the amplification efficiency with the formula: E = (10^(-1/slope) - 1) * 100. The host-specific primers targeting GAPDH showed an amplification efficiency of approximately 110%, while the virus-specific primers achieved an efficiency of ~107%. Once the primer efficiencies were established, the time-course experiment was performed. In this time-course experiment, HZ-AM1 cells were infected with HzNV-1 at a multiplicity of infection (MOI) of 5, with triplicate samples collected at hourly intervals from 1 hour post-infection (hpi) to 10 hpi. Total DNA was extracted at each time point using the DNeasy Blood & Tissue kit (Qiagen). To determine the timing of viral DNA replication initiation, qPCR measurements from 2 hpi to 10 hpi were compared to the baseline value measured at 1 hpi. For both experiments, relative viral DNA levels were calculated using the Pfaffl method (1), which is an enhanced version of the ΔΔCt method that accounts for varying primer efficiencies. The formula used was: Ratio = (E_target / E_reference) ^ (ΔCq_reference - ΔCq_target). For all qPCR measurements, a total of 60 ng DNA was used as template per individual well. The calculated fold changes were visualized in a bar plot with their respective standard errors of the mean (Figure S1).

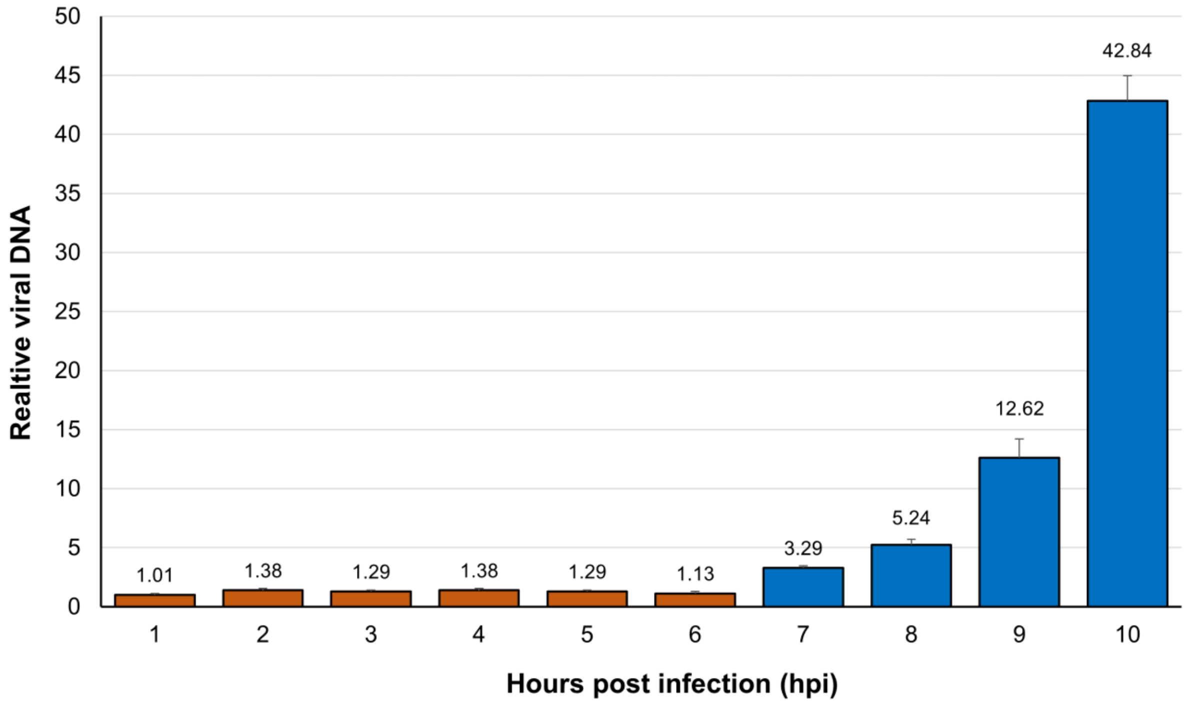

Fig. S4. Bar graph illustrating the fold changes in relative viral DNA levels measured using quantitative PCR (qPCR) across different time points of HZ-AM1 cells I with HzNV-1 (MOI = 5). The housekeeping gene of the host, *gapdh*, was used as an internal reference to normalize the data, while the targeted viral gene region was *vlf-1*. The Pfaffl method was employed to calculate the fold changes based on the difference to the reference time point 1 hpi (earliest

infection time point). The y-axis represents the fold change, with error bars indicating the standard error of the mean.

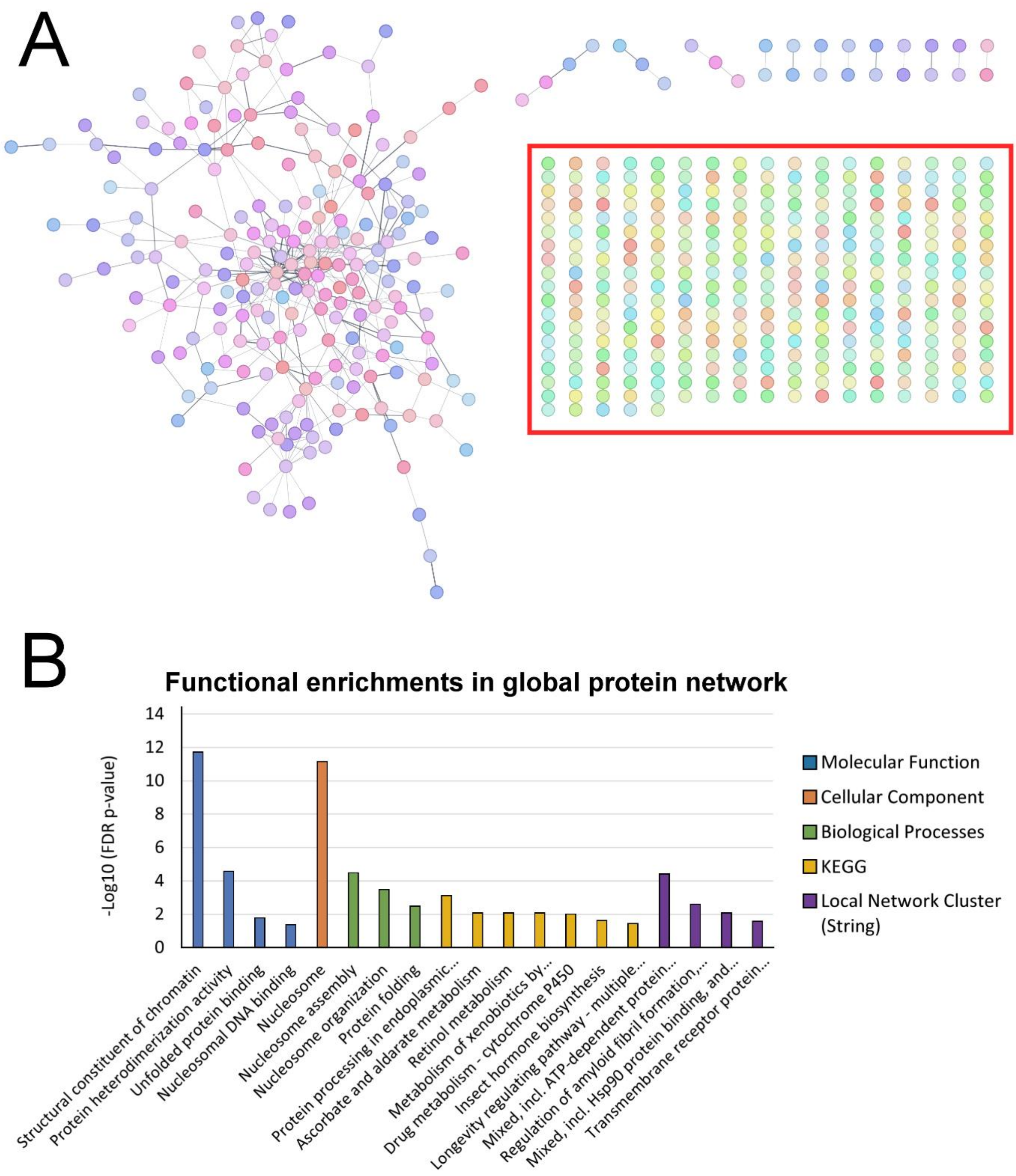

Fig. S5. Protein-protein interaction (PPI) network analysis of all differently expressed genes (DEGs) during HzNV-1 infection. (A) Generated network before clustering, showing the global PPIs. Shared functional enrichment terms among nodes are color-coded, and the thickness of the node-connecting lines represent the confidence of these interaction. The nodes in the red frame showed no supported interactions with any of the other proteins. The PPI was generated

using the STRING database (3) and the supporting software Cytoscape (4). (B) Functionally enriched gene annotation terms of the global protein network visualized in a bar chart against their respective negative log10 transformed false discovery rate (FDR) p-values.

References

1. Pfaffl MW. Relative quantification. Real-time PCR: Taylor & Francis; 2007. p. 89-108.
2. Kariyawasam H, Su S, Voogd O, Ritchie ME, Law CW. Dashboard-style interactive plots for RNA-seq analysis are R Markdown ready with Glimma 2.0. NAR Genomics and Bioinformatics. 2021; 3 (4): lqab116.
3. Szklarczyk D, Kirsch R, Koutrouli M, Nastou K, Mehryary F, *et al.* The STRING database in 2023: protein–protein association networks and functional enrichment analyses for any sequenced genome of interest. Nucleic acids research. 2023; 51 (D1): D638-D46. DOI: 10.1093/nar/gkac1000
4. Shannon P, Markiel A, Ozier O, Baliga NS, Wang JT, *et al.* Cytoscape: a software environment for integrated models of biomolecular interaction networks. Genome Res. 2003; 13 (11): 2498-504. DOI: 10.1101/gr.1239303
